# Time-of-day perception in paintings

**DOI:** 10.1167/jov.24.1.1

**Published:** 2024-01-02

**Authors:** Cehao Yu, Mitchell J. P. Van Zuijlen, Cristina Spoiala, Sylvia C. Pont, Maarten W. A. Wijntjes, Anya Hurlbert

**Affiliations:** 1 Perceptual Intelligence Laboratory, Faculty of Industrial Design Engineering, Delft University of Technology, Delft, The Netherlands; 2 Cognitive Informatics Lab, Department of Intelligence Science and Technology, Graduate School of Informatics, Kyoto, Kyoto University, Japan; 3 Neuroscience, Institute of Biosciences, Faculty of Medical Sciences, Newcastle University, Newcastle, UK

**Keywords:** image statistics, time-of-day perception, chromatic properties, art history, light

## Abstract

The spectral shape, irradiance, direction, and diffuseness of daylight vary regularly throughout the day. The variations in illumination and their effect on the light reflected from objects may in turn provide visual information as to the time of day. We suggest that artists’ color choices for paintings of outdoor scenes might convey this information and that therefore the time of day might be decoded from the colors of paintings. Here we investigate whether human viewers’ estimates of the depicted time of day in paintings correlate with their image statistics, specifically chromaticity and luminance variations. We tested time-of-day perception in 17th- to 20th-century Western European paintings via two online rating experiments. In Experiment 1, viewers’ ratings from seven time choices varied significantly and largely consistently across paintings but with some ambiguity between morning and evening depictions. Analysis of the relationship between image statistics and ratings revealed correlations with the perceived time of day: higher “morningness” ratings associated with higher brightness, contrast, and saturation and darker yellow/brighter blue hues; “eveningness” with lower brightness, contrast, and saturation and darker blue/brighter yellow hues. Multiple linear regressions of extracted principal components yielded a predictive model that explained 76% of the variance in time-of-day perception. In Experiment 2, viewers rated paintings as morning or evening only; rating distributions differed significantly across paintings, and image statistics predicted people's perceptions. These results suggest that artists used different color palettes and patterns to depict different times of day, and the human visual system holds consistent assumptions about the variation of natural light depicted in paintings.

## Introduction

Painters have long been attuned to real-world properties that are relevant to the perceiver (Mamassian, 2008) and have developed effective techniques to represent everyday scenes in pictorial space (Cavanagh, 2005). While not aiming for physical accuracy, their depictions often contain invariants (Gibson, 1971) or perceptual shortcuts (van Zuijlen, Lin, Bala, Pont, & Wijntjes, 2021) that support the viewer's understanding of the scene. As such, paintings provide a rich source of image features that vision scientists can use to better understand human visual perception.

Analysis of these features has largely focused on aesthetic preference (Hayn-Leichsenring, Lehmann, & Redies, 2017; Nakauchi & Tamura, 2022) or material properties, such as transparency (Sayim & Cavanagh, 2011), translucency (Wijntjes, Spoiala, & de Ridder, 2020), gloss (Di Cicco, Wijntjes, & Pont, 2019), or velvetiness (Di Cicco, Van Zuijlen, Wijntjes, & Pont, 2021). Less well explored is how perceivers may also infer more abstract yet ecologically important dimensions from paintings, such as time of day or weather. For these, painters may use explicit cues such as human activities, shadow length, or sun position. Yet other image features, independent of pictorial content, may powerfully convey the time of day. Here we examine the relationship between low-level image statistics, in particular the distribution of chromaticities and luminances, in paintings and the depicted time of day.

In representational paintings, painters deploy pigment on canvas to capture the effects of light interacting with surfaces in the scenes they depict. Variations in chromaticity and luminance across the image, induced by complex material–light interactions, may contain essential information about three-dimensional (3D) structure (Bloj, Kersten, & Hurlbert, 1999; Ruppertsberg, Bloj, & Hurlbert, 2008); these painted patterns may in turn evoke perceptions of 3D shape and surface color. Luminance shading defines fundamental elements of volume and space (Arnheim, 1974) and provides cues to the location and orientation of objects and the direction of the light (Carbon & Pastukhov, 2018; Kartashova, de Ridder, te Pas, Schoemaker, & Pont, 2015). In paintings, cast shadows may indicate the light source position, even when simplified beyond physical plausibility (Casati, 2008; Cavanagh, 2005; Ostrovsky, Cavanagh, & Sinha, 2005). Cast shadow lengths (Hagen, 1976) might give an additional indication of the time of day.

**Figure 1.**
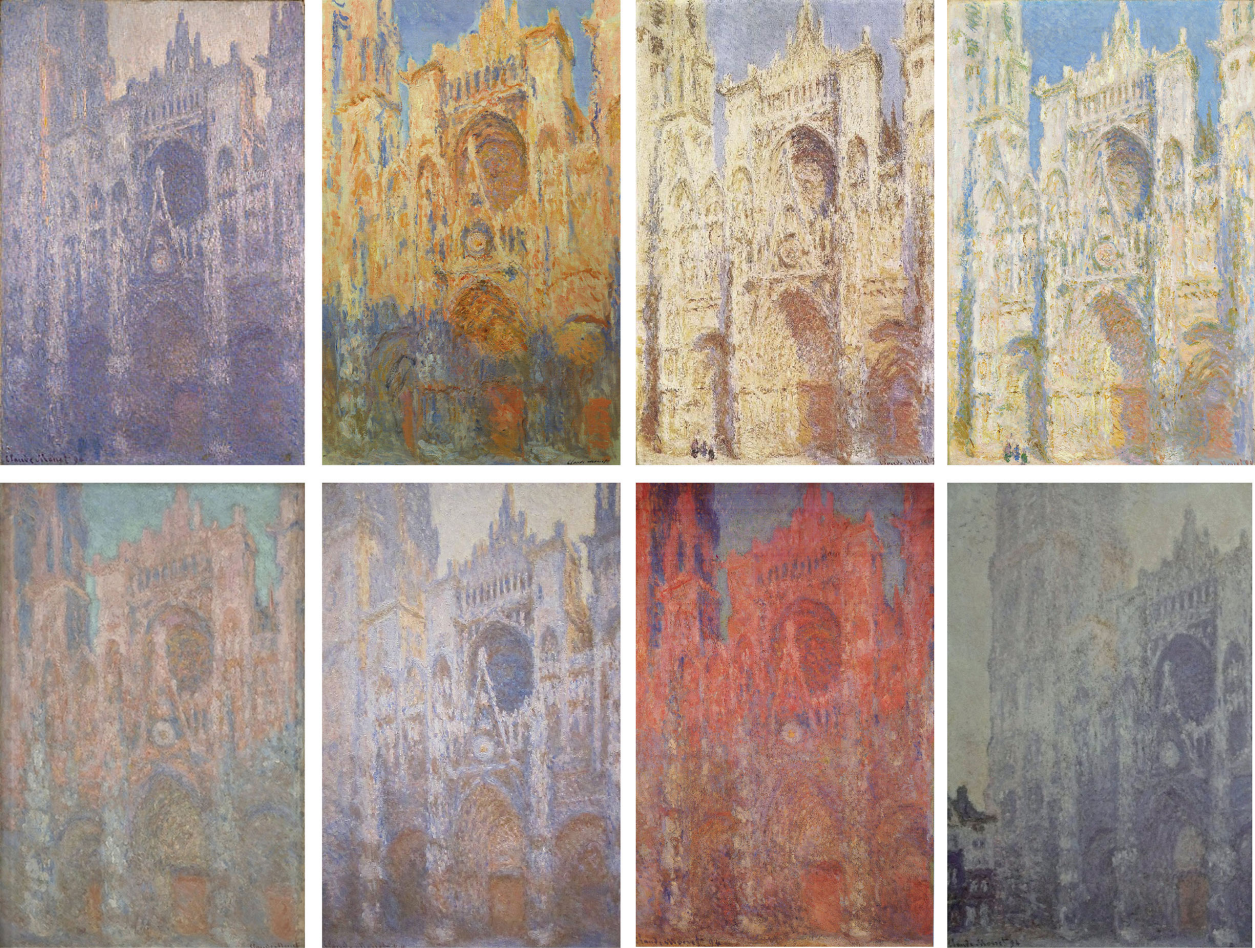
Claude Monet's series paintings of Rouen Cathedral (1892–1894). The paintings capture the façade of the Rouen Cathedral at different times of the day and year under various weather conditions. Monet's depictions effectively amplify the changes in color appearance that a typical color-constant viewer would perceive and reflect his own personal experiences and visual capabilities. Downloaded from Wikimedia Commons.

Chromatic content might also be used in paintings to depict time of day. J.M.W. Turner's pair of paintings, *The Morning* *After the Deluge* and *The Evening of the Deluge*, seem by their titles and content to demonstrate an association between color and time of day, as well as weather. *The Morning* *After the Deluge* features a cyclone of brilliant colors, converging on yellow and white, evoking a sunny day. In contrast, *The Evening of the Deluge* features blackness encircling a gray-blue core, suggesting a stormy night. The paintings not only pay explicit homage to Goethe's color theory (Goethe, 1810) but also express an implicit rule about the depiction of time. In his series paintings of Rouen Cathedral, Claude Monet painted different still moments of the cathedral in markedly different color palettes, titling them with different times of day. In *Rouen Cathedral, Facade (sunset)* (Figure 1, upper row second), the orangish glow of the solid stone partially covered by a crisp bluish shadow under a blue sky creates a visual impression distinct from *The Portal of Rouen Cathedral in Morning Light* (Figure 1, upper row first), in which the smoothly shadowed, inarticulate façade dissolves into the background sky. The questions we pose are whether in deploying such chromatic cues painters are capturing natural variations in illumination over the course of the day and season and whether people consistently read time of day from these cues.

**Figure 2.**
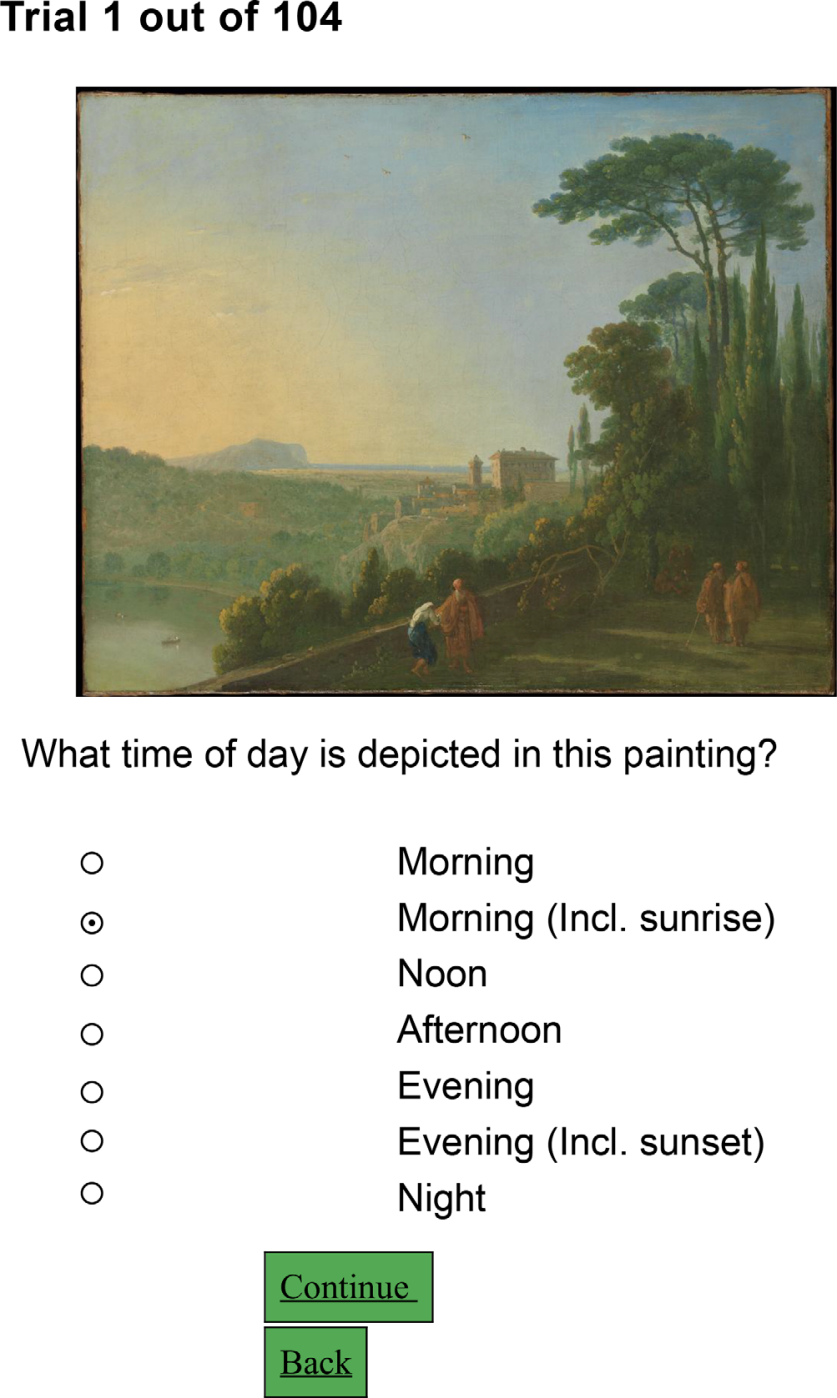
The interface of Experiment 1.

**Figure 3.**
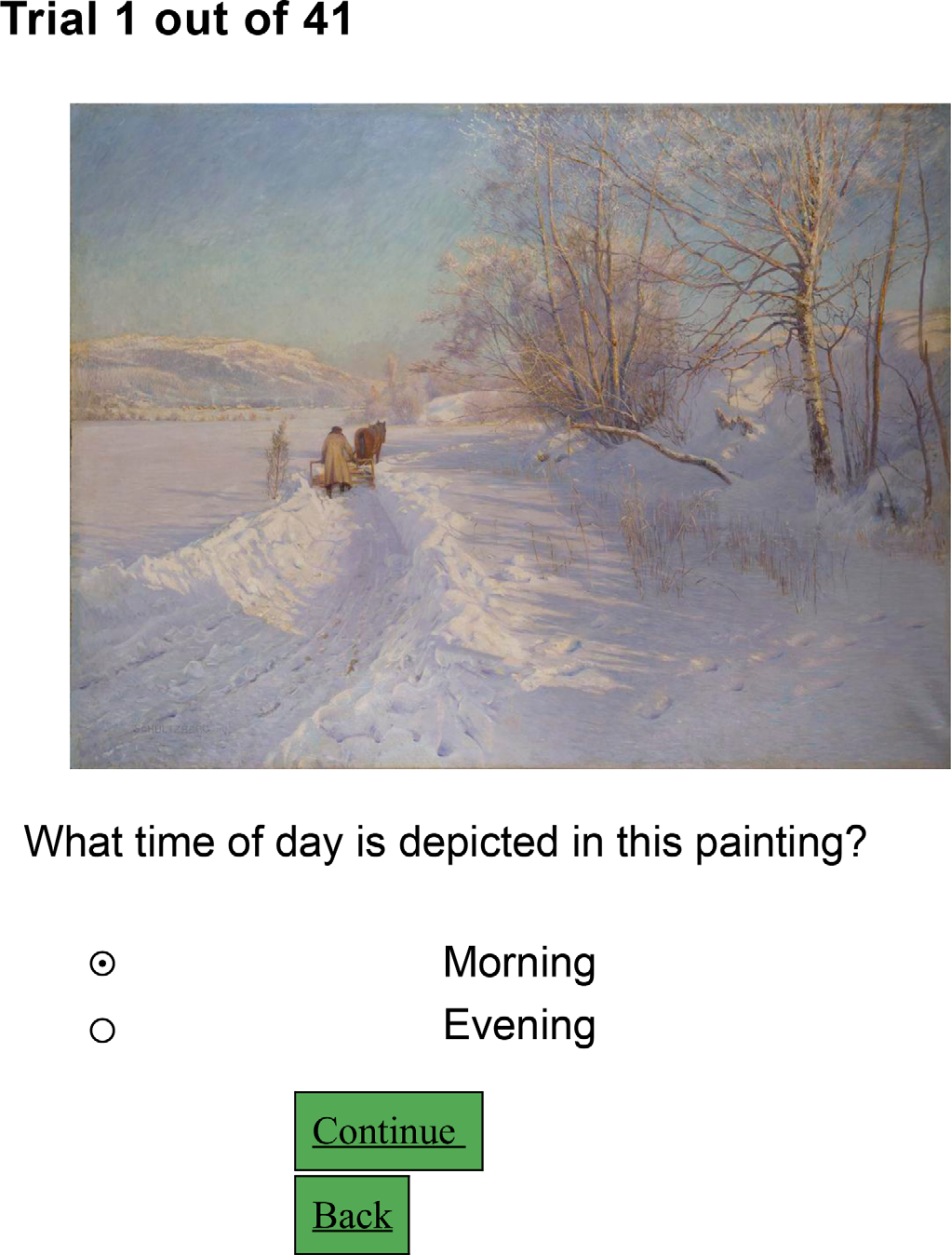
The interface of Experiment 2.

### Characteristics of terrestrial illumination

The spectrum, direction, and diffuseness of natural illumination in the terrestrial world change over the course of a day and across seasons due to interactions between sunlight, atmospheric conditions, the environment, and anthropogenic light. Diurnal illumination, or daylight, is the total light originating from the sky and sun after sunrise and before sunset, while crepuscular illumination, or twilight, is the sum of the skylight and artificial anthropogenic light when the sun disk is below the horizon. Nocturnal illumination, in the third phase of the day, is commonly provided by moonlight, starlight, and light pollution between astronomical dusk and astronomical dawn.

Solar elevation is the main determinant of the illuminance and chromaticity of natural illumination (Minnaert, 1993; Smith, 2005). The illumination is more diffuse and less intense during early morning and late afternoon compared to midday (Hoeppe, 2007; Mardaljevic, 2019). This is due to the longer path sunlight travels through the atmosphere, allowing for greater scattering at lower elevations. Additionally, low sun angles also result in lower illuminance on upward-oriented surfaces, as the amount of light reaching a surface is proportional to the cosine of the angle of incidence. The spectral composition of illumination also changes throughout the day, with the proportion of short to long wavelengths in light that reaches the earth decreasing as the wavelength-dependent (Rayleigh) scattering increases, skewing the transmitted beam more toward reddish hues at sunrise and sunset.

In line with the idea that the human visual system has evolved to be attuned to the characteristics of natural illumination (Morgenstern, Geisler, & Murray, 2014; Pastilha & Hurlbert, 2022; Shepard, 1992), here we suggest that the variations in chromaticity and illuminance of natural illumination, and their effect on the light reflected from objects, might also influence painters’ choices of color palettes and, in turn, people's interpretations of the depicted environment.

### Sunrise–sunset asymmetry

If solar elevation were the sole determinant of their appearance, morning and evening skies should be indistinguishable. Gombrich acknowledged this ambiguity when he wrote of Corot's work (Gombrich, 1976), “Corot softens the shadow of the fallen tree and of the goose, thus convincingly suggesting the mellow light of morning or evening.” Beurs, however, in his observations from the 17th century, noted that although the techniques used to depict sunrise and sunset may be similar, there are distinct differences in the color palette and temperature of the sky (Beurs, 1692). He observed that sunrise often features cooler colors and more mists, while sunset has warmer colors and holds the warmth of the day. Beurs's observations may be explained by the diurnal temperature and humidity cycle that affects the amount and type of light scattering by atmospheric particles.

As the sun sinks toward the horizon during sunset, the evening air begins to cool, causing larger water molecules to gather in the atmosphere and scatter the long-wavelength component of the sunlight, turning the sky an orangey-red hue (Panorgias, Kulikowski, Parry, McKeefry, & Murray, 2012). In the morning, the air, still laden with overnight moisture and coolness, is denser with smaller water particles. Airlight, the light scattered toward the viewer from atmospheric particles, becomes brighter with longer pathlengths, and its hue is determined by the size of the scattering particles (Koenderink, 2010; Koschmieder, 1924; Narasimhan & Nayar, 2002). Morning mist, haze, and fog generate a bluish airlight with a milky, diffuse quality (Deutsch, 1991; Minnis, Mayor, Smith, & Young, 1997; Pechony, Price, & Nickolaenko, 2007; Rickel & Genin, 2005).

In the present study, we set out to investigate whether human observers can estimate depicted time of day in paintings and, if so, whether these perceptions may be related to image statistics. We hypothesize that the image statistics of paintings contain information about the characteristics of terrestrial illumination and that human observers use this statistical regularity to judge the time of day depicted in a painting. To test this hypothesis, we conducted two rating experiments with 17th- to 20th-century paintings. Experiment 1 involved participants viewing digital reproductions of paintings and selecting the time of day depicted from seven options. The observation that bimodal distributions resulted for certain paintings, with some participants rating them as morning and others as evening, motivated Experiment 2. Its aim was to examine whether observers were able to distinguish between morning and evening in paintings when given only those choices, using a stimulus set with metadata to provide “ground truth.”

## Methods

We conducted two online experiments, recruiting participants via Amazon Mechanical Turk (AMT). In Experiment 1, participants were presented with digital reproductions of paintings and asked to choose the time of day depicted from seven options: sunrise, morning, noon, afternoon, evening, sunset, and night (see Figure 2). In Experiment 2, participants were asked to select between morning or evening (see Figure 3). We analyzed the perceptual data in relation to image statistics to better understand whether humans use image statistics to judge the time of day depicted in paintings.

### Image data set

The images of paintings were downloaded from online open-access data sets, including the Materials in Painting (MIP) data set (van Zuijlen et al., 2021; https://materialsinpaintings.tudelft.nl) and the National Gallery (NG) data set (https://nationalgallery.org.uk/paintings). These data sets were chosen because they display a wide diversity of natural outdoor scenes under a variety of illumination conditions.

### Stimuli

In order to focus specifically on the role of image statistics related to light and color and their relationships with people's perceptions of the time of day in paintings, we selected primarily outdoor scenes that would be influenced by natural light. We also chose paintings that lacked explicit social or contextual cues, such as human activities, which might easily indicate the depicted time of day.

In Experiment 1, we chose 104 high-resolution digital images of 17th- to 20th-century oil paintings (see Figure A3). This collection comprised 50 from the MIP data set and 54 from the NG data set. For eight paintings out of the total selection, the title contained information about the depicted time of day (e.g., *Evening at Medfield, Massachusetts* by George Inness). We also selected four paintings from the NG data set as catch trials. These four catch trials clearly depicted nighttime scenes and were identified as nighttime depictions according to their titles or metadata (e.g., *A River* *Near a Town, by Moonlight* by Aert van der Neer). The metadata consist of information about a painting that is not necessarily provided by the painter but rather by curators or other art experts with art historical knowledge and expertise.

In Experiment 2, we chose a new set of 90 digital images, distinct from those in Experiment 1, featuring 17th- to 20th-century paintings from the MIP data set (refer to Figure A3). The titles of these paintings provided cues to the time of day represented in each scene: sunrise (10 paintings), morning (17 paintings), sunset (36 paintings), and evening (27 paintings). To standardize the stimuli, we resized the images to 1,000 pixels along the longer dimension, while preserving the original aspect ratio.

All paintings reproduced within this article are available under open access at a Creative Commons Zero (CC0) or Creative Commons Attribution-Noncommercial (CC BY-NC) 4.0 license. The complete list of all paintings used within this study, including those reproduced in this article, is available in Data Set 1 (Yu, 2023).

### Observers

A total of 112 unique (Experiment 1, *n* = 51; Experiment 2, *n* = 61) participants were recruited via the AMT platform. Each agreed to the informed consent before data collection. Data collection was approved by the Human Research Ethics Committee of the Delft University of Technology and adhered to the ethical guidelines of the Declaration of Helsinki. All observers were naive to the purpose of the experiments.

Previous experience with AMT recruitment has suggested that data might be noisy due to a small but considerable portion of participants who appear to perform poorly in experiments (Di Cicco et al., 2021; van Zuijlen, Pont, & Wijntjes, 2020). We thus set an exclusion criterion in Experiment 1 to automatically remove participants who scored below an 80% correct rate for the catch trials (detailed below). In total, 25 participants were removed this way. The exclusion was performed prior to data analysis.

### Procedure and task

We used a similar procedure for both Experiments 1 and 2. Experiment 1 consisted of seven alternative choices, and Experiment 2 comprised two alternative choices. Participants were informed that they would be presented with images of paintings and that they would indicate the time of day in each trial. After each labeling, participants had to press the continue button for the subsequent trial. Participants were also allowed to go back and redo the previous trials. The trials were randomized across participants.

In Experiment 1, there were 109 trials per observer. Experiment 2 was composed of three blocks, each containing 41 trials. Block 1 involved 21 observers, while Blocks 2 and 3 had 20 observers each. Within each block, there was no repetition of stimuli. Among three blocks, there were 70 unique stimuli. Thirteen stimuli were used in all three blocks, and seven stimuli were used in each of two blocks.

### Image analysis

Our hypothesis is that painters capture the variation in illumination and reflected light from scenes over the course of a day, and therefore the paintings will vary in their luminance and chromatic content according to the time of day they depict. We hypothesize that participants will be able to discern and interpret this content and that its statistical characteristics will predict people's perception of the time of day. We therefore examined whether the image statistics of paintings predict participants’ time-of-day ratings.

The images in this data set were downloaded as photographic jpegs or pngs and displayed directly without further transformation in the participants’ Internet browser windows. To model the color appearance of the paintings as viewed by each participant and from this calculate the image statistics of the paintings, we assume that for each participant, (a) the display calibration characteristics and (b) the external viewing conditions stayed constant throughout the experiment. For each session, the same color transformation from RGB pixel values to color appearance will therefore apply across the entire image data set. For the main analyses, we use the sRGB color space model as the basis for that transformation. sRGB is the widely adopted standard color model for image display on monitors and the web. It defines chromaticities for the RGB primaries, based on original CRT phosphors, and a nonlinear transfer function between input digital value (*v*) and output intensity (*I*), with *I* = *v^γ^* and *γ* = 2.2. Using the sRGB model, we calculated the color appearance of the paintings displayed by converting RGB pixel values into chromaticity and luminance coordinates in CIE standard color spaces and derived further image statistics from these. Although the sRGB model might not perfectly predict color appearance for each participant's display, it is the optimal transformation for approximating the average appearance, and it also allows for consistent comparison and analysis of the image statistics across all images in the data set. We show in further analyses that calculated image chromaticities and luminances are strongly correlated across alternative white points for the sRGB color transformations (Figure A5). Additional analyses using laboratory screen calibrations confirm the main results reported below.

The color appearance attributes and image statistics, as described in detail in the Appendix, were computed for our analysis. To the reader less well versed in colorimetry: We are essentially converting colors from screen-dependent coordinates (i.e., the RGB values of the digital images) to screen-independent, standardized color coordinates. We then employed these standardized color metrics as input for a principal component analysis (PCA) to reduce the dimensionality of the data.

#### Color specifications and appearance metrics

Assuming the sRGB model and a default white point of D65, we calculated 1,931 CIE XYZ values for each pixel and, from these, CIELAB and LCH, according to standard formulae, as detailed in the Appendix. Furthermore, we directly converted the sRGB pixel values to cone, rod, and melanopic photoreceptor activations. We used the cone fundamentals specified by Stockman et al. (Stockman & Sharpe, 2000; Stockman, Sharpe, & Fach, 1999), the melanopsin curve by Lucas et al. (2014), and the scotopic curve by Crawford (1949) to compute the scotopic irradiance. We combined precomputed spectra for sRGB primaries, which have minimal roundtrip errors (as established by Mallett & Yuksel, 2019), to generate the corresponding spectrum for given sRGB pixel values.

For brightness and lightness measures, we used the CIE Y tristimulus value (termed luminance in the analyses below) and CIELAB L* (termed lightness below).

For chromaticity measures, we used CIELAB a* and b*, hue (calculated from CIELAB a* and b*, as in the Appendix), saturation (calculated from CIELAB a*, b* and L*, as in the Appendix), and chroma (calculated from CIELAB a* and b*, as in the Appendix). Because the chromaticity of daylight may be summarized by its correlated color temperature (CCT), which is the temperature of the black-body radiator with the nearest chromaticity on the Planckian locus in CIE 1960 (*u*, *v*) space, we therefore also convert CIE XYZ values for each pixel into CCT (in Kelvin).

#### Statistical measures

For each of the luminance, lightness, and chromaticity metrics above, we calculated descriptive statistics (max, min, mean, variance, and skewness) of their pixel value distributions for each image. For the luminance and the blue channel in sRGB, we also calculated RMS contrast. See the Appendix for formal definitions.

In addition, we derived further image descriptors relating to interactions between chromaticity and luminance across each image:

**Color difference at maximum luminance difference:** To summarize overall contrast, including both luminance and chromatic contrast, we calculated the color difference between the brightest and darkest pixels in each image, using the CIE ∆*E* 2000 color-difference formula (∆*E*00). The CIE ∆*E* 2000 color-difference formula (Luo, Cui, & Rigg, 2001), based on CIELAB coordinates, is the recommended standard for computing color differences that are perceptually uniform across color space.
**Luminance-weighted CCT:** The luminance-weighted CCT is calculated for each pixel as the product of the pixel's CCT and its corresponding luminance. Luminance thereby serves as a weighting factor, reducing the contribution of darker pixels and increasing the contribution of brighter pixels. Effectively, this weighting recognizes the greater salience of brighter pixels in chromaticity perception.
**Pixel-wise luminance–chromaticity correlations:** We also calculated the correlation between pixel luminance and chromaticity measures (CIELAB b*, saturation, and chroma) (Nakauchi & Tamura, 2022) within each image using Pearson's correlation coefficients.
**Luminance–chromaticity image distance:** We determined the mean distance between the luminance and each of the three chromatic channels (CIELAB b*, saturation, and chroma). By calculating the difference between pixel values at corresponding locations within the respective channels, we obtained a measure of the pixel-wise chromaticity-luminance relationship for individual images.

#### Image airlight color

To provide further insight into the atmospheric conditions conveyed by the variation in chromaticity and luminance within each image, we computed an estimate of airlight appearance using the dark channel prior method (He, Sun, & Tang, 2009). This approach identifies and removes areas where at least one spectral band is darker than the others before averaging the remaining pixel values that correspond to clusters with the highest average luminance and lowest chromatic saturation. The resultant airlight color has been shown to relate to the presence of scattered light in hazy images. For more information, please refer to the Appendix.

### Statistical analysis

To quantify participants’ responses, we used chronological scaling systems. In Experiment 1, we used two scaling systems: The first assigned scores of 1 to 7 to seven rating categories in chronological order: sunrise, morning, noon, afternoon, sunset, evening, and night. This 7-point scale was used for the PCA to examine the distribution of the original categories and identify potential overlaps. For all other analyses, including the correlational analyses and linear model prediction, we used a 4-point scale, merging into single categories (1) morning and sunrise, (2) noon and afternoon, and (3) evening and sunset, and keeping night in the final category. These were assigned scores of 1, 3, 5, and 7, respectively. This scale took account of potential ambiguity in the perceived chronology of sunrise and sunset relative to morning and evening. In Experiment 2, we simplified the scale to two categories, assigning 0 to morning and 1 to evening.

We used three measures for data analysis: (a) the mean score (“mean time of day”): the sum of all the scores divided by the number of scores, providing an overall measure of the perceived time of day for each painting; (b) the proportional score per category: the ratio between the total count of scores for a particular category and the total number of scores, or the proportion of scores that fall into each category; and (c) categorical score: the mode of the scores given by participants for a particular painting, indicating the most frequently perceived time of day for that painting.

Two types of analyses were conducted to investigate the relationship between participants’ responses and image statistics. The first consisted of independent correlation analyses on each of the image metrics for both experiments using mean time-of-day scores. The second used the principal components extracted from the image metrics as predictors in a multiple regression analysis to model the mean time of day. The accuracy and quality of the model were evaluated using the Akaike information criterion (AIC). Results with a *p*-value less than 0.05 were considered statistically significant.

## Results

### Experiment 1

**Figure 4.**
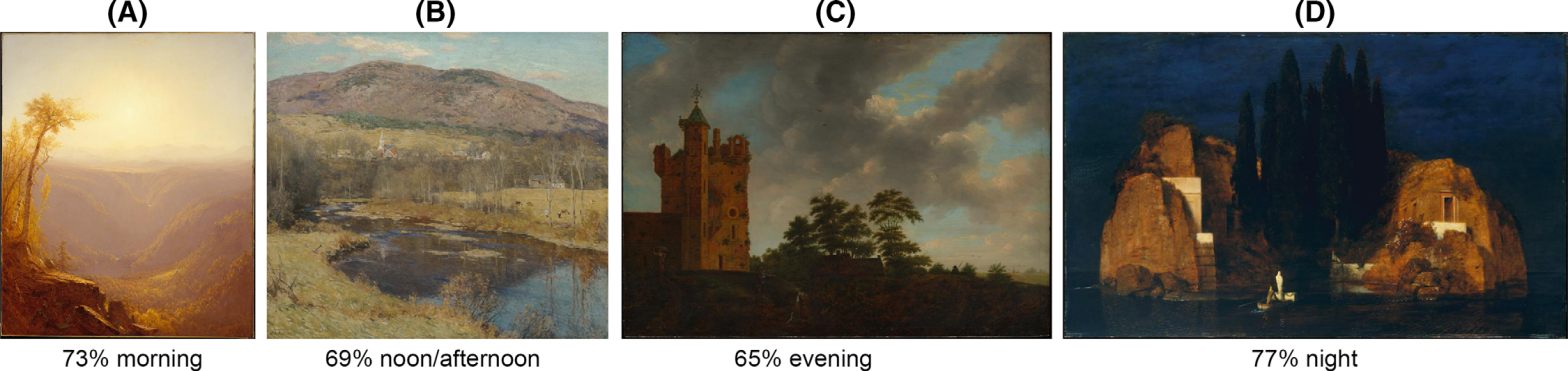
Sample paintings with high-consistency ratings. (**A**) Sanford Robinson Gifford, *A Gorge in the Mountains (Kauterskill Clove)*, 1862. (**B**) Willard Metcalf, *The North Country*, 1923. (**C**) Emanuel Murant, *The Old Castle*, 1642–1700. (**D**) Arnold Böcklin, *Island of the Dead*, 1880. Downloaded from the online repository of the Metropolitan Museum of Art, New York.

**Figure 5.**
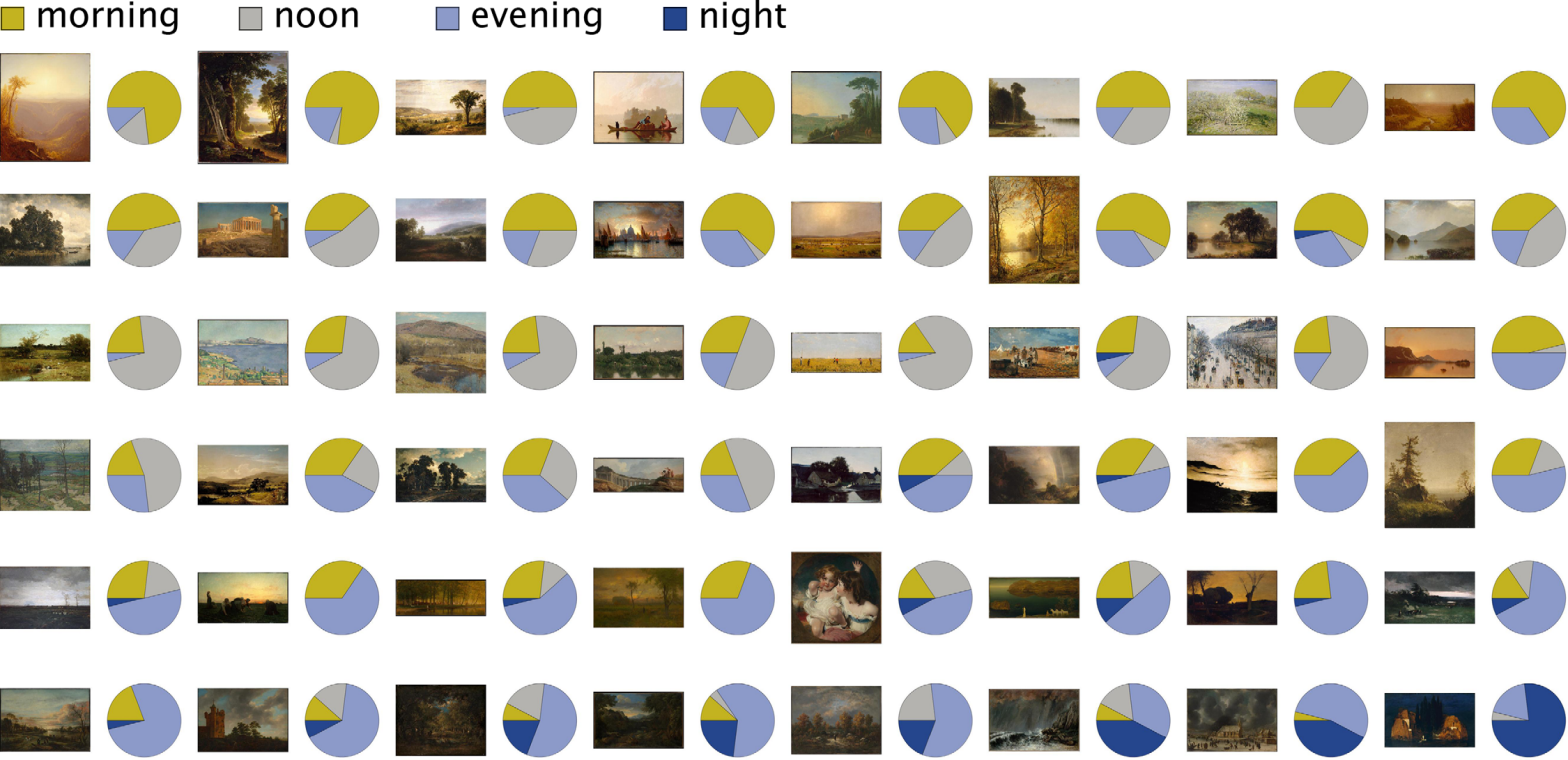
Percentages of population responses for the four merged categories for 48 of the MIP subset of paintings, Experiment 1.

The results indicate that people are readily able to assess the time of day depicted in paintings. Observers’ ratings varied significantly across paintings (analysis of variance [ANOVA]; *F*(103, 2600) = 15.23, α = 0.05/5356, *p* < 0.00001). There was no significant difference between results with and without the data that did not pass the catch-trial selection criterion (Mann–Whitney test, *p* = 0.82). For individual images, response distributions varied. For a small proportion of images, responses were concentrated in a single category, with maximum categorical scores of 77% for morning, 81% for noon/afternoon, 77% for evening, and 100% for nighttime (see Figure 4 for examples of paintings with high interobserver consistency). Other images exhibited a more evenly distributed mixture of ratings, with approximately 12% of paintings having a bipolar distribution of morning–evening ratings (see Figure 5 for response category distributions for the MIP paintings subset). The evident ambiguity of this latter group provided partial motivation for Experiment 2. Despite this variation in response distributions, a substantial 24.9% of pairwise comparisons between paintings (equating to 1,335 pairs) still presented significant differences (Bonferroni corrected).

#### Image analysis

**Figure 6.**
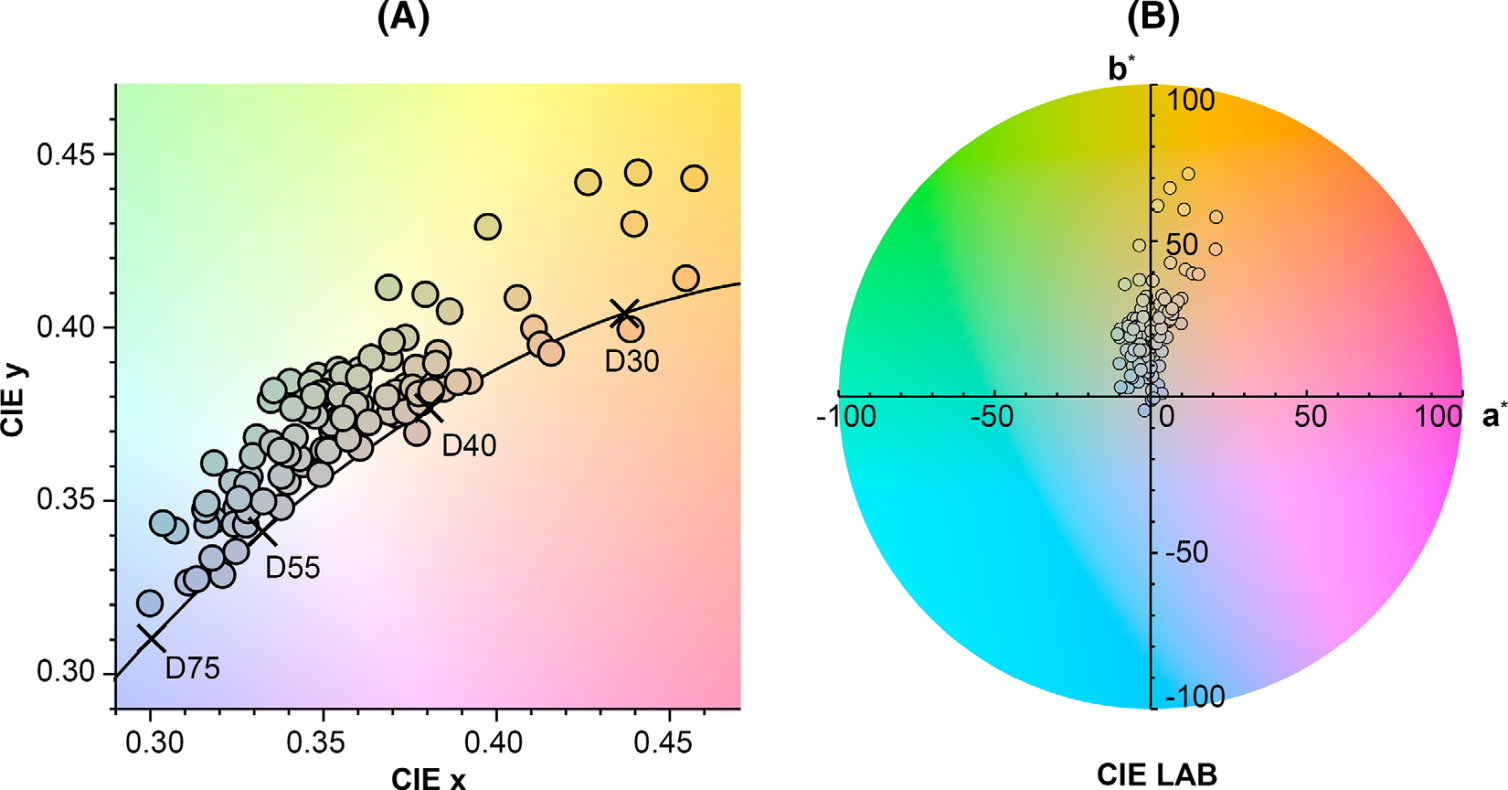
(**A**) Mean image chromaticities in the CIE xy plane for paintings, one disk per image. Disk colors approximately represent image chromaticity. The black line indicates the daylight locus; the locations of D30, D40, D55, and D75 are marked. (**B**) The CIE LAB plane at a lightness level (L*) of 0.8; one disk per painting.

**Figure 7.**
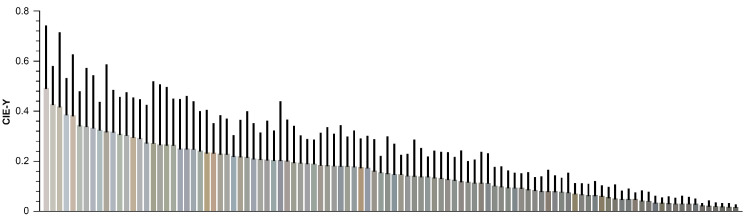
Illustration of mean image luminance for each painting, depicted by individual bars arranged in descending order from highest to lowest luminance. The colors of the bars represent mean image chromaticity, while the black line on top of the bars shows the standard deviation.

**Figure A1.**
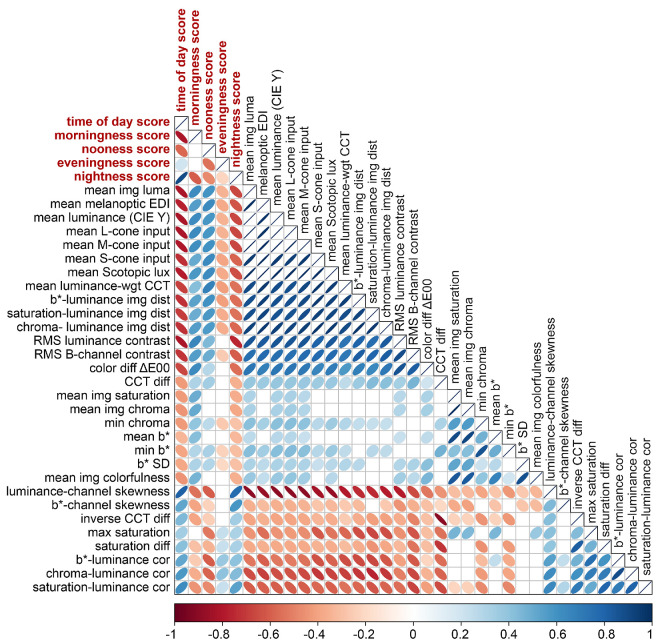
Results of Experiment 1. Correlation matrices of human rating and image statistics are represented by ellipses that vary in thickness and color. Thinner ellipses indicate a stronger correlation, while fatter ellipses indicate a weaker correlation. The elongation of the ellipses shows the direction of correlation, with red indicating negative correlation, blue indicating positive correlation, and white indicating zero correlation. Only correlation coefficients that have a significant effect at *p* < 0.05 are included in the matrix cells.

For each image, we calculated 30 image statistics that capture the chromaticity and luminance variations, as well as their relationships, within and across the image set (see Methods for detailed descriptions and Figure A1 for the complete list). The mean CIE chromaticities of all paintings are shown in Figure 6, with one disk representing each painting. The chromaticities tend to cluster along the daylight locus, varying from blueish to orangish. Almost all lie above the daylight locus, with a positive *D_uv_* value indicating a greenish shift. This chromatic relationship holds across different white points (Figure A5). Figure 7 presents the mean luminance of the paintings, providing additional descriptive statistics for the image set.

**Figure 8.**
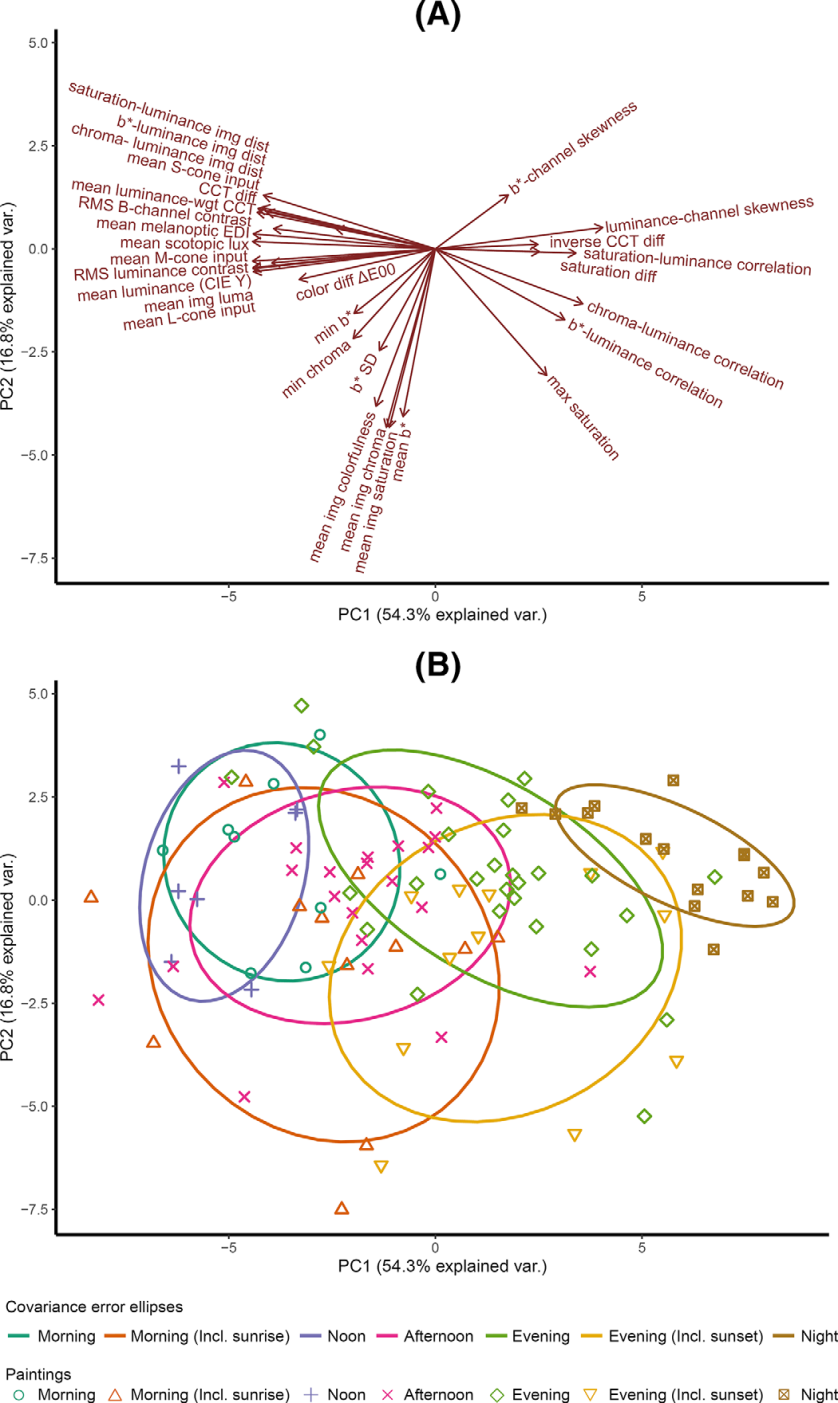
Visualization of the first two dimensions of PCA. (**A**) Factor loadings of 30 image statistics, with text labels, and the red vectors indicating the factor loadings of the original dimensions. (**B**) The covariance ellipses that were fitted for each time-of-day class, in which each point represents one of the 104 paintings and is color-coded based on its perceived time-of-day classification.

We conducted a PCA to uncover the underlying dimensions of the space, because the image statistics are not independent of each other (see Figure A1 and Table A1). We extracted five principal components (PCs), as defined by eigenvalues greater than 1 (Table A2). The first two components (Dim1–PC1 on the horizontal axis and Dim2–PC2 on the vertical axis) accounted for 71.1% of the variability in the image statistics data, as visualized in Figure 8. Adding a third, fourth, or fifth component captured 81.7%, 86.8%, and 90.4% of the variability, respectively. In the PC space, there were three distinct clusters of factor loadings, indicated by the red arrows, which were distributed on the positive and negative sides of the horizontal axis and the negative side of the vertical axis (Figure 8A).

It is important to emphasize that the orientation of PCs is arbitrary; their signs can be flipped, and their interpretation remains the same. For ease of discussion and interpretation, we chose a specific orientation to present our results. Principal component 1 (PC1) is highly negatively loaded with measures such as luminance, cone, rod, and melanopic photoreceptor activations; contrast; luminance-weighted CCT; and chroma–luminance image distance but positively loaded with luminance–channel skewness, which negatively correlates with the other measures. These relationships indicate that brighter images also exhibit higher contrast and are skewed toward brighter pixel values, consistent with depictions of strong daylight. PC1 also captures the relationship between luminance and chromaticity within images via its negative loading with luminance-weighted CCT and chroma–luminance distance. Whereas PC1 thus encapsulates luminance-related measures, PC2 instead represents chromaticity-related measures: Mean image saturation, mean image chroma, mean b*, and mean image colorfulness all load negatively on PC2. CCT, inverse CCT difference, and saturation differences loaded the third principal component (PC3). The highest loadings on the fourth and fifth principal components (PC4, PC5) came from minimal image b* and image b* standard deviation, respectively.

#### Relationship between observer responses and image metrics

To evaluate whether the above image metrics predict time-of-day ratings, we performed correlations (visualized in Figure A1) and found that all 30 image statistics were significantly correlated with mean time-of-day scores (*p* < 0.0001 for all). We report correlations where the absolute value of *r* is greater than 0.6, namely, positive correlations for luminance skewness (*r* = 0.82) and saturation−luminance correlation (*r* = 0.62), as well as negative correlations for mean image luminance (*r* = −0.81), mean image luma (*r* = −0.81), mean melanopsin input (*r* = −0.78), mean L-cone input (*r* = −0.82), mean M-cone input (*r* = −0.81), mean S-cone input (*r* = −0.73), mean rod input (*r* = −0.79), RMS luminance contrast (*r* = −0.81), RMS contrast (B channel) (*r* = −0.71), b* standard deviation (*r* = −0.72), ∆E00 (*r* = −0.67), mean luminance-weighted CCT (*r* = −0.69), b*-luminance image distance (*r* = −0.72), saturation−luminance image distance (*r* = −0.69), and chroma−luminance image distance (*r* = −0.73).

We further calculated the PC1 and PC2 coordinates of each image. Assigning each image its categorical score (as defined above) on the 7-point scale, we calculated the mean image PC coordinates and covariance matrix for each category. From the eigenvalues and eigenvectors of the covariance matrices, we derived the major and minor axes of the covariance ellipses for each category. These are illustrated in Figure 8B, with the ellipses enclosing 68% of the data points within each category, corresponding to one standard deviation from the mean for normally distributed data. The covariance ellipses reveal that sunrise and sunset cover the largest area, while night covers the smallest area. Sunrise and sunset loaded on combined components, while other time-of-day groups primarily loaded on the first component. The factor loadings for the first five components are shown in Table A1.

To predict the mean time-of-day rating, which spans from morning to night across four combined categories, we applied a multiple linear regression analysis using the components extracted from our PCA. By applying the forward technique, we added one extra component as a predictor at a time. Accordingly, we computed five candidate models and compared them using AIC model selection (Burnham & Anderson, 2002). A lower Akaike weight can be interpreted as a higher probability that a certain model performs best. Table 1 shows the statistical summary for all the candidate models. We selected the model with the lowest Akaike weights, which included only PC1 and PC2. The equation for the best-fitting line is

(1) $$\begin{array}{ccc} & & mean\;time\;of\;day\;rating\\ & & \;=0.583+0.034PC1+0.017PC2\; \end{array}$$

**Table 1.** Model summary. *Notes*: Dependent variable: mean time-of-day rating.

					Change statistics				
*R*	*R* ^2^	Adjusted *R*^2^	Standard error of the estimate	AIC	*R* ^2^ change	*F* change	*df*1	*df*2	Sig. *F* change
0.840^a^	0.706	0.703	0.09046	−202.675	0.706	245.379	1	102	0.000
0.872^b^	0.760	0.755	0.08218	−221.675	0.054	22.599	1	101	0.000
0.872^c^	0.761	0.754	0.08245	−220.010	0.001	0.322	1	100	0.571
0.876^d^	0.767	0.758	0.08173	−220.878	0.007	2.769	1	99	0.099
0.876^e^	0.768	0.756	0.08205	−219.129	0.001	0.236	1	98	0.628

a Predictors: (Constant), PC1.

b Predictors: (Constant), PC1, PC2.

c Predictors: (Constant), PC1, PC2, PC3.

d Predictors: (Constant), PC1, PC2, PC3, PC4.

e Predictors: (Constant), PC1, PC2, PC3, PC4, PC5.

**Figure 9.**
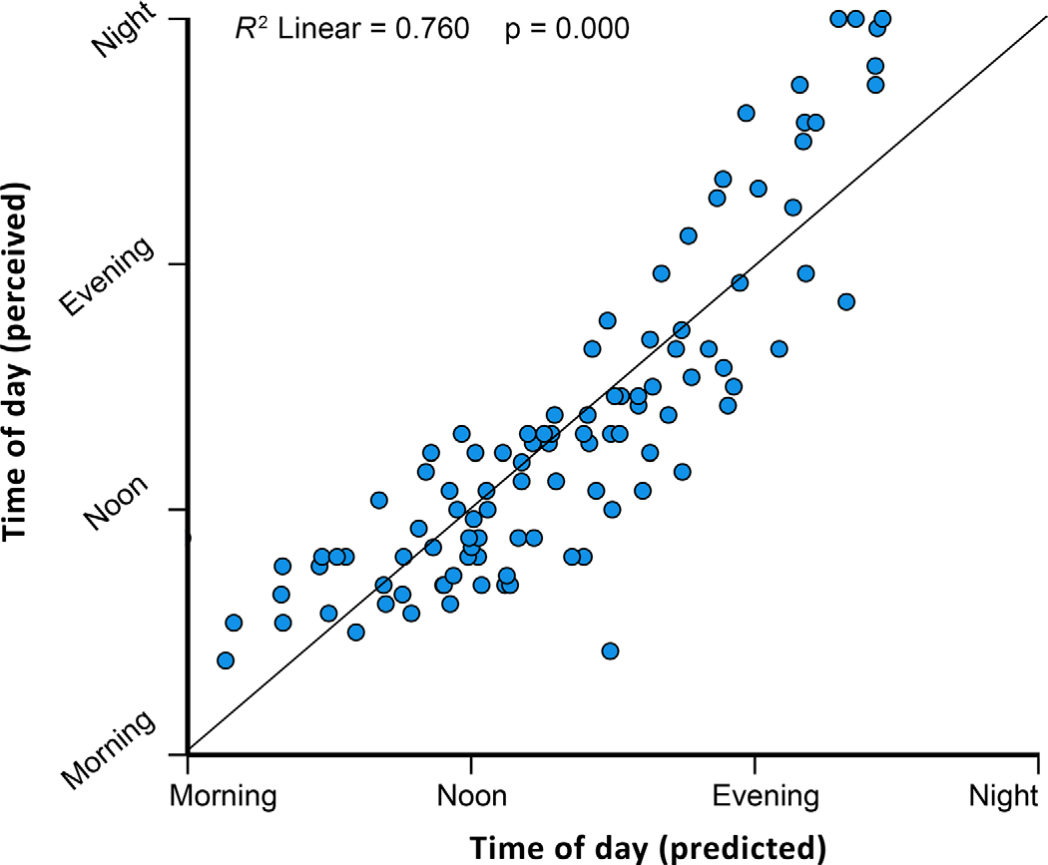
Scatterplot comparing the average perceived time-of-day scores (y-axis) to the predicted time-of-day scores (x-axis) based on the linear model established with the four merged time-of-day category scores.

This model explained (*R*^2^) 76% of the perceived time-of-day variance (see Figure 9).

### Intermediate discussion

In Experiment 1, we found that the mean image chromaticities of the paintings were distributed over a large range, close to and slightly above the daylight locus. This implies that the mean image chromaticities of the stimuli ranged from so-called warm to cool daylight, with a slight green shift. The green shift might be due to a large portion of landscape features in the painting content. Perceived time of day correlated with various image statistics incorporating luminance and chromatic information. Multiple linear regressions of extracted principal components resulted in a two-dimensional predictive model that explained 76% of the variance in time-of-day perception. People seem to use assumptions about the variation in brightness and color of natural light depicted in paintings to infer the time of day, yet with large interindividual differences. Some paintings were perceived consistently as morning, noon, evening, or night, while for many paintings, we found mixtures of three or four ratings. Some paintings had ratings split almost evenly between morning and evening. This observation partially motivates Experiment 2.

### Experiment 2

**Figure 10.**
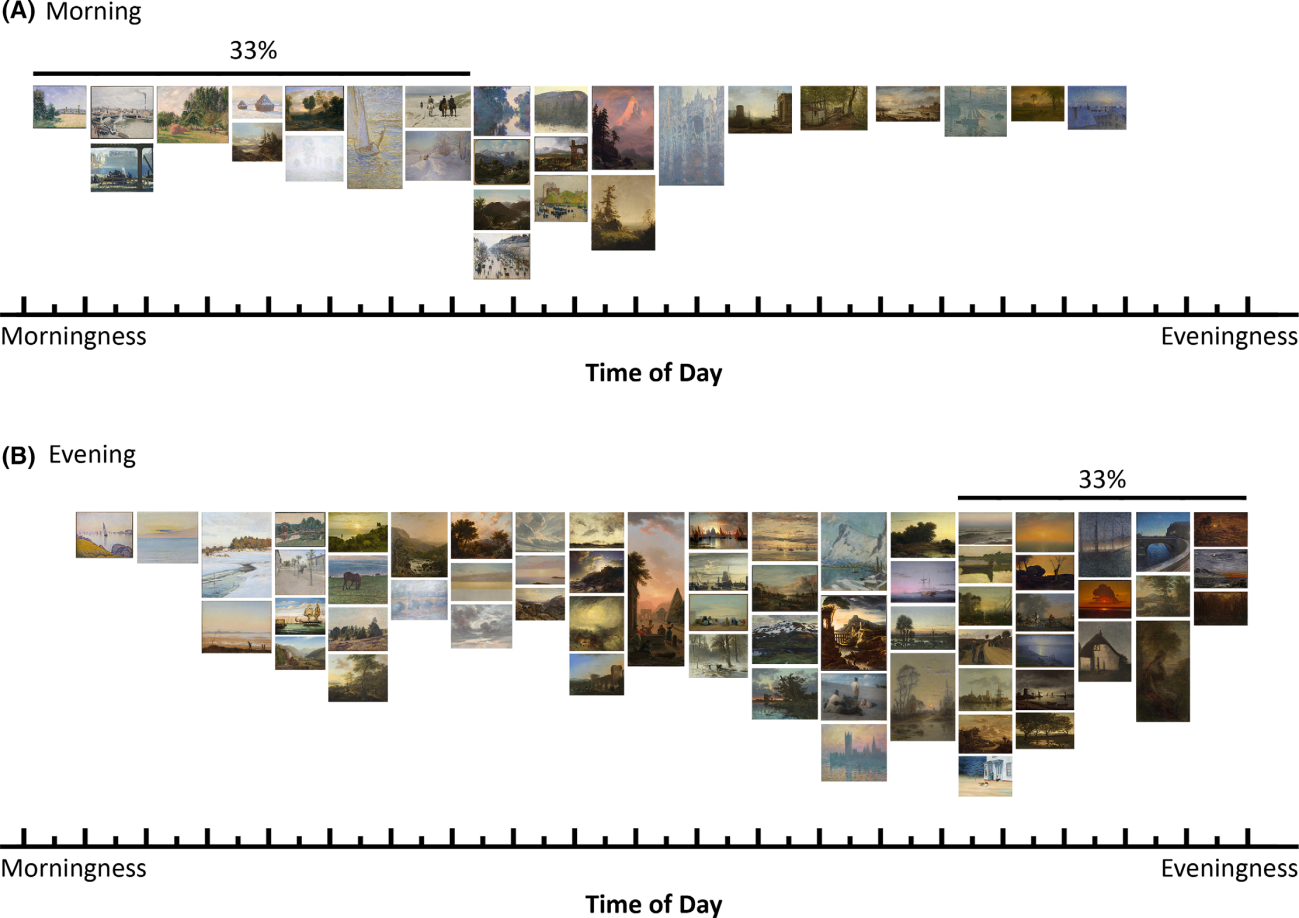
Morningness−eveningness perception in Experiment 2. Digital images of paintings from the MIP data set were ordered according to the average time-of-day score. The order from left to right corresponds to a progression from morningness to eveningness. (**A**) Metadata-indicated morning or sunrise scenes (27 total). (**B**) Metadata-indicated evening or sunset scenes (63 total). The top 33% rated morning and evening paintings are marked by the solid line.

In Experiment 2, we investigated further whether observers could discriminate specifically between morning and evening in paintings that explicitly portrayed these times of day. Grouping metadata-labeled “morning” and “sunrise” paintings together into the metadata-morning category and “sunset” and “evening” into the metadata-evening category, we found that observers’ ratings were indeed significantly different for the two categories (Mann–Whitney test, *p* = 0.000). Figure 10 illustrates the results, with the paintings ordered according to average participant ratings, from morningness (left) to eveningness (right). Metadata-morning paintings are illustrated in the upper panel and metadata-evening paintings in the lower panel. Although paintings in both categories cover a large time span, they clearly cluster toward either side. A chi-square test on morning versus evening counts (defined by the threshold value 0.5) further demonstrated significant agreement between the metadata and the participants' labeling, with χ²(1) = 13.235 and *p* < 0.001.

**Figure 11.**
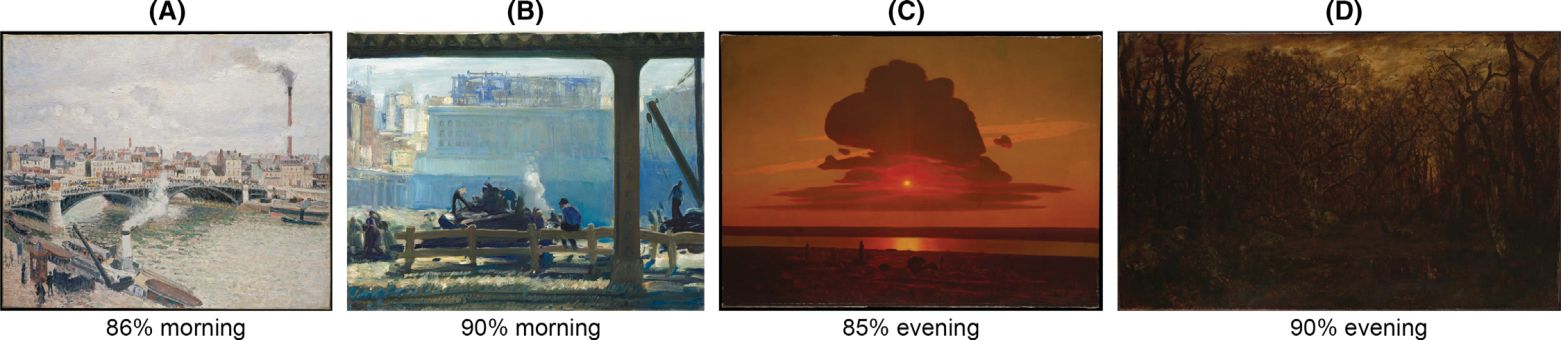
Sample paintings and their ratings. (**A**) Camille Pissarro, *Morning, An Overcast Day, Rouen*, 1896. (**B**) George Bellows, *Blue Morning*, 1909. (**C**) Arkhip Ivanovich Kuindzhi, *Red Sunset on the Dnieper*, 1905–1908. (**D**) Théodore Rousseau, *The Forest in Winter at Sunset*, c. 1846–c. 1867. Downloaded from the online repository of the Metropolitan Museum of Art, New York.

Although there was a significant difference in observer ratings across the images (ANOVA; α = 0.05/4005, *p* << 0.00001, *F*(89, 4235) = 14.5139), there was also a large range of ratings distributions across individual paintings, with some morning paintings being rated as morning by 95% of the participants and some evening paintings as evening by 100% of participants. Overall, 27.9% of all painting pairs had significantly different ratings distributions (Bonferroni corrected). Figure 11 illustrates several paintings with highly consistent interparticipant ratings.

#### Relationship between observer responses and image metrics

**Figure A2.**
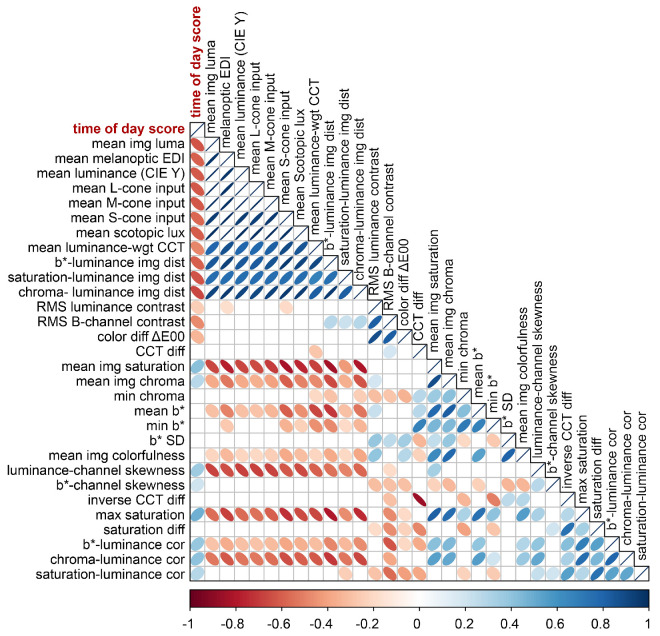
Results of Experiment 2. Correlation matrices of human rating and image statistics are represented by ellipses that vary in thickness and color. Thinner ellipses indicate a stronger correlation, while fatter ellipses indicate a weaker correlation. The elongation of the ellipses shows the direction of correlation, with red indicating negative correlation, blue indicating positive correlation, and white indicating zero correlation. Only correlation coefficients that have a significant effect at *p* < 0.05 are included in the matrix cells.

**Figure A3.**
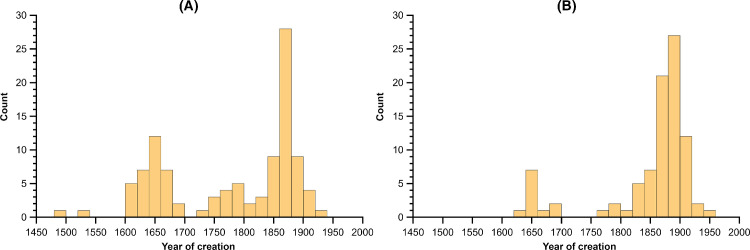
Histogram of years of creation for paintings used in Experiment 1 (**A**) and Experiment 2 (**B**).

As for Experiment 1, we computed the set of 30 image statistics for each painting and conducted single linear regressions between these and the time-of-day rating. The resulting correlation matrix is visualized in Figure A2. Several image statistics, including Min chroma, mean b*, min b*, b* SD, mean image colorfulness, CCT difference, inverse CCT difference, and saturation difference, were no longer significantly correlated with the observers’ ratings. The correlations between the human ratings and mean image saturation and chroma reversed in direction, with higher saturation now associating with higher eveningness ratings.

**Figure 12.**
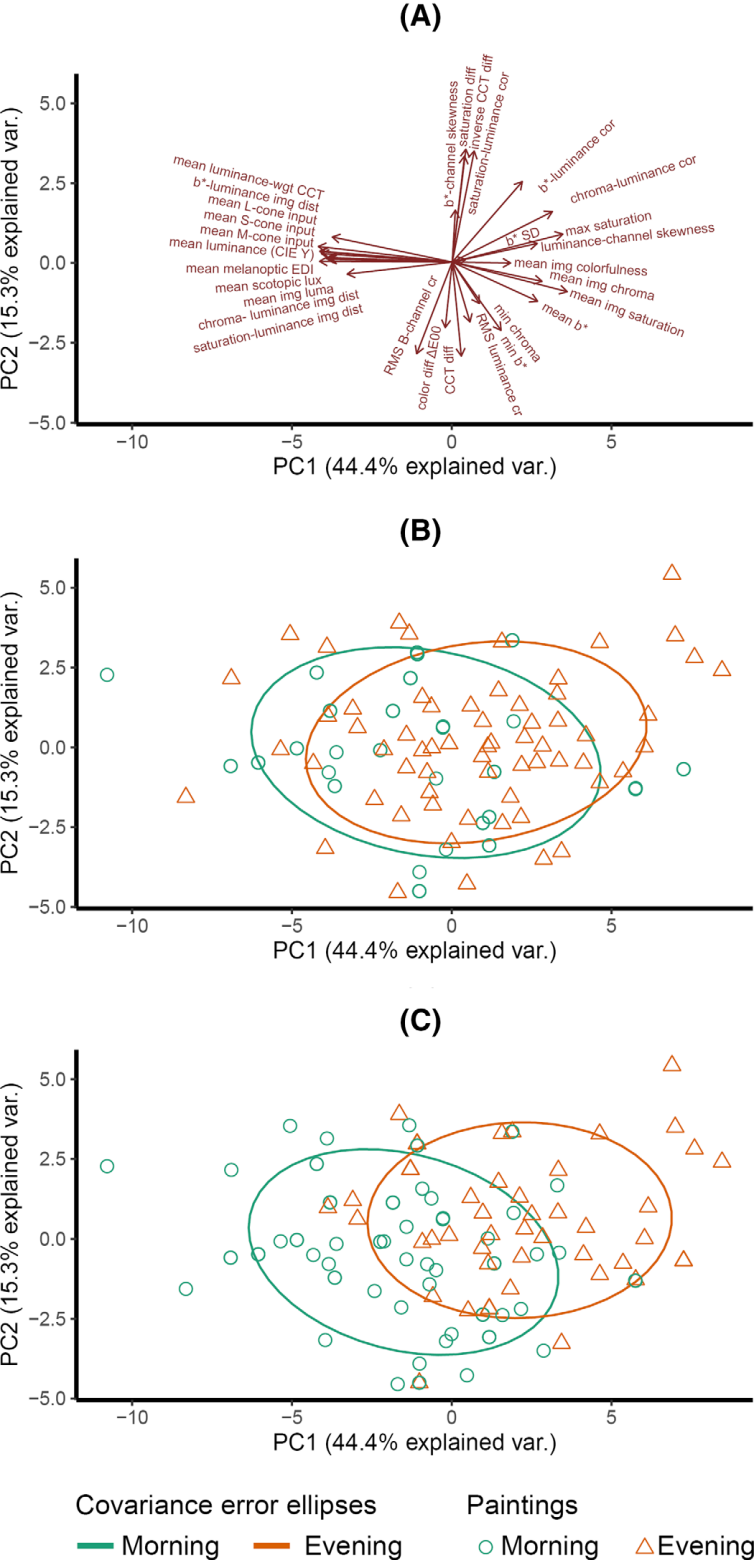
Biplot visualizations of the first two principal components. (**A**) Red arrows indicate the factor loadings of all image statistics. (**B**) Morning and evening paintings based on metadata classification, with points representing the 90 paintings and colors indicating the classification. (**C**) Morning and evening paintings based on observers’ ratings classification, with points and colors representing the classification. Ellipses were fitted to enclose 68% of the data points within each category.

A principal components analysis on the 30 image statistics yields the results illustrated in Figure 12. Subpanel (A) displays the factor loadings, while subpanels (B) and (C) display the positioning of the metadata-classified and observer-classified morning and evening paintings, respectively, in the multidimensional image feature space. The observer-classified categories are based on classifications selected by a majority of the participants (e.g., observer-classified morning paintings were rated as morning by more than 50% of observers). We used the same method as in Experiment 1 to fit ellipses designed to enclose 68% of the data points within each category.

Notably, the clusters for morning and evening paintings based on metadata classification showed significant overlap (Figure 12B), while the clusters based on observer classification demonstrated less overlap (Figure 12C). This difference in overlap is primarily attributable to the reduction in the size of the ellipses, rather than shifts in their positions. Despite these differences in overlap, the shapes of the covariance ellipses remained largely consistent across both classification methods.

The factor loadings for the PCs identified in this analysis were similar to those found in Experiment 1 on the negative side of the horizontal axis but different for the remaining factor loadings (Figure 12A). This and the reversal of certain correlations compared with Experiment 1 is probably due to the narrower range of paintings in this data set, consisting only of morning and evening paintings, relative to the broader time-of-day selection in Experiment 1.

PC1 coordinates were significantly different between metadata-morning and metadata-evening (Mann–Whitney test, *p* = 0.000), but PC2 coordinates were not (Mann–Whitney test, *p* = 0.294). Yet for observer-classified morning versus evening paintings, both PC1 and PC2 differed significantly (Mann–Whitney test, *p* = 0.000 and *p* = 0.005, respectively). Thus, our results suggest that observers perceive more pronounced differences in depicted time of day than the metadata specify.

**Figure 13.**
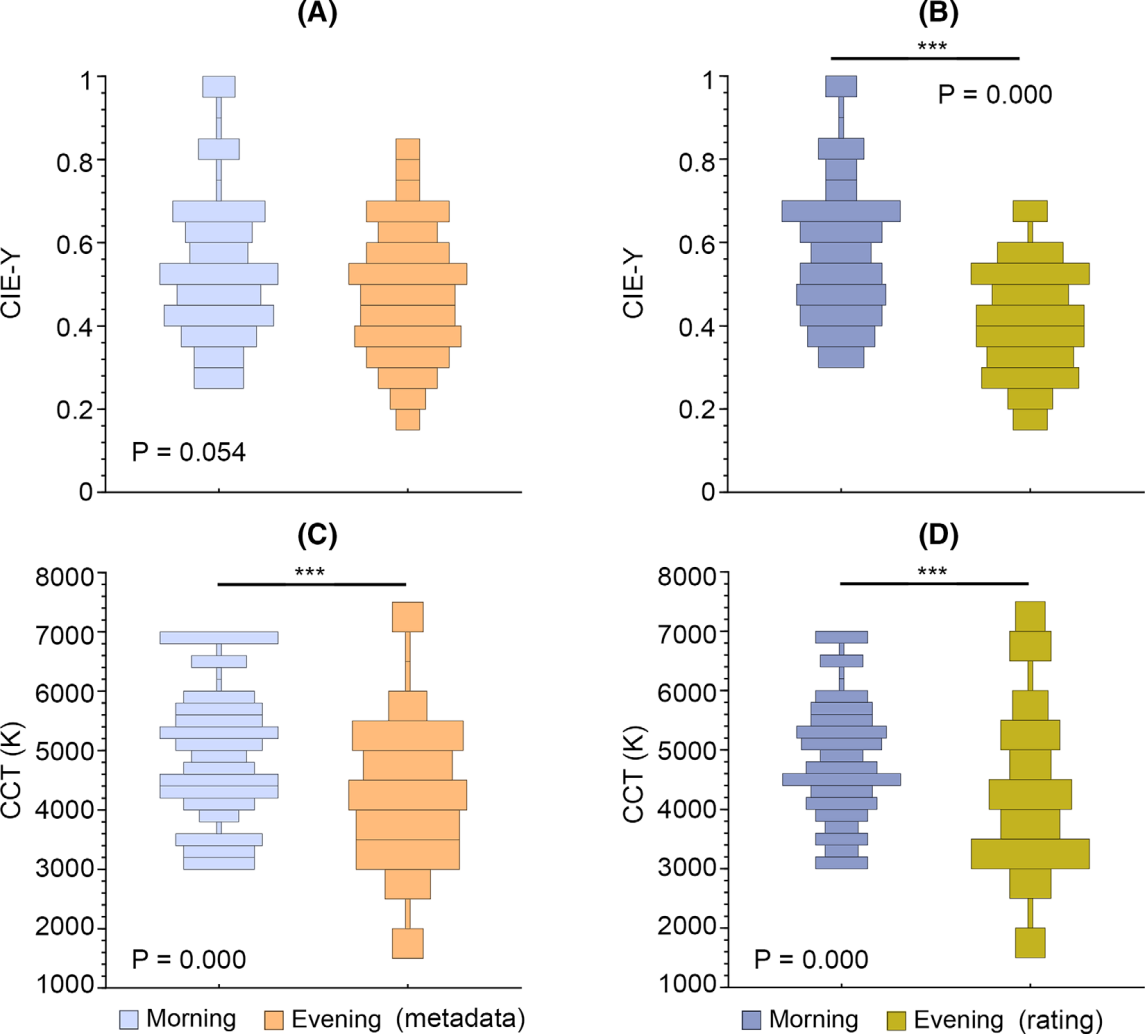
Distribution plots of mean image luminance (CIE Y) and chromaticity (CCT). (**A**, **C**) Metadata classification. (**B**, **D**) Observers’ rating classification. ***Statistically significant differences (Mann–Whitney test, *p* < 0.0001).

To probe the image factors underlying observers’ time-of-day ratings, we compared the distributions of luminance-related and chromaticity-related image statistics for both observer-classified and metadata-classified morning and evening paintings. Figure 13 shows the CIE Y and CCT distributions for all paintings, with observer classifications shown in the right plots and metadata classifications in the left plots. The analysis revealed that while there were no significant differences between mean image luminance for metadata-indicated morning and evening paintings (Figure 13A), there was a significant difference based on observer classifications (Figure 13B). The mean image chromaticity (CCT) differed significantly between morning and evening paintings for both metadata and observer classifications (Figures 13C, D).

**Figure 14.**
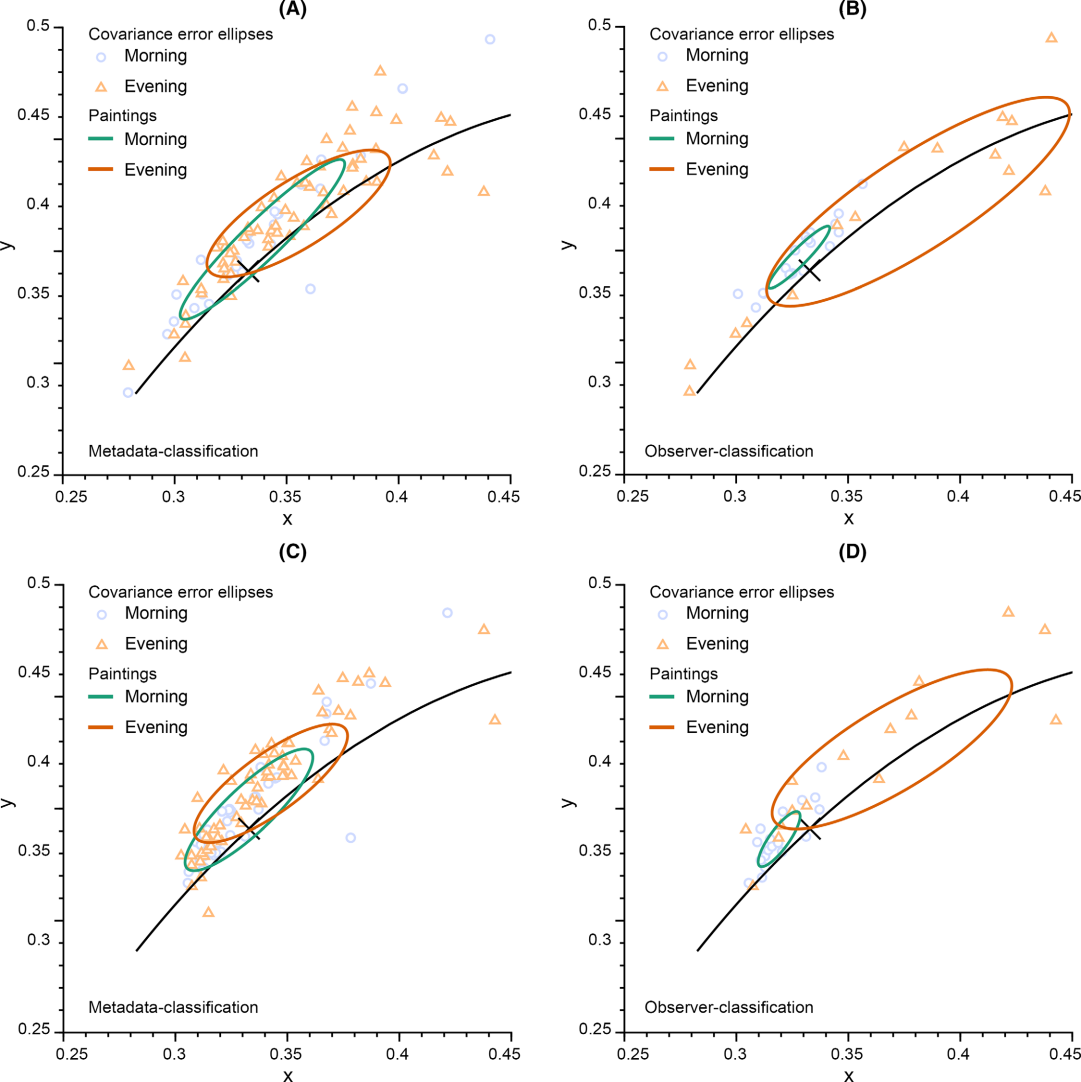
Mean image chromaticities and airlight chromaticities of paintings plotted in the CIE xy plane. (**A**, **B**) Mean image chromaticities based on metadata and observer ratings, respectively, with the top 33% rated morning and evening paintings included in panel (**B**). (**C**, **D**) Airlight color chromaticities based on metadata and observer ratings, respectively, with the top 33% rated morning and evening paintings included in panel (**D**). The black line indicates the daylight locus, and the diagonal cross indicates D55, serving as a reference for the chromaticity values.

To explore the influence of chromaticity on perceptions of morning versus evening, beyond the one-dimensional metric CCT, we analyzed the CIE x,y distributions of the third most extreme observer-rated morning versus evening paintings. (These paintings are indicated by the 33% line in Figure 10.) For all paintings, the mean image chromaticities generally follow the daylight locus, as shown in Figures 14A, B. For both metadata-classified and observer-rated morning paintings, the mean chromaticities cluster near D50, much more tightly for the observer-rated paintings. The mean chromaticities of evening paintings cover a large range of the daylight locus but are weighted toward the warm end, with a larger covariance error ellipse for the observer-rated than metadata-classified paintings. (Mean image CCTs: for metadata-classified images: morning 5233K, evening 4692K; for observer-rated images: morning 5686K, evening 3945K.)

Mean airlight chromaticities, which correlate with both the chromaticities of diffuse ambient illumination (Koenderink, 2010) and with the amount of haze (Narasimhan & Nayar, 2002), generally shift to cooler chromaticities for both metadata-rated (Figure 14C) and observer-rated (Figure 14D) paintings relative to the mean image chromaticities, as expected. (Mean airlight CCTs: for metadata-classified images: morning 5482K, evening 5116K; for observer-rated images: morning 6131K, evening 4305K.)

The exception to this is the observer-classified evening paintings, for which the mean airlight chromaticity is slightly shifted to warmer colors. Overall, for both mean image and airlight chromaticities, the morning paintings tend to be bluer than the evening paintings.

### Intermediate discussion

In Experiment 2, we set out to investigate whether there are notable differences in the way that artists depict morning and evening and whether observers can use these differences to identify morning or evening. To this end, we selected a set of paintings with metadata indicating whether they depict a morning or evening scene and asked observers to classify the paintings as either morning or evening. The mean image chromaticities of morning paintings were close to neutral white, while those of evening paintings varied from warm white to cool white, with a high frequency of warm white occurrences. The airlight chromaticities of morning paintings were on average bluer than those of evening paintings. These differences were found to be larger between observer-classified morning and evening paintings than between metadata-classified morning and evening paintings. This suggests that, overall, there may be different statistical regularities between morning and evening depictions and that people are able to use those to differentiate between morning and evening.

## General discussion

We studied the time-of-day perception using 17th- to 20th-century oil paintings. In Experiment 1, we collected human ratings on the time of day depicted in paintings. We collected both quantitative image statistics and qualitative perceptual data. These showed systematic correlations yielding insights into how the time of day can be perceived and predicted. Variance in the data was high, but statistical regularities in the human data correlated with regularities in the image characteristics. We found that “morningness” correlated with bright, high-contrast, saturated, and brighter blue/darker yellow images and “eveningness” with darker, low-contrast, desaturated, and brighter yellow/darker blue images. This finding shows that image statistics related to light and color in paintings reflect the characteristics of terrestrial illumination and can be used by people to perceive the depicted time of day. Specifically, luminance and chromaticity were found to be the most effective predictors of perceived time of day. The desaturation association with eveningness might also be related to the Hunt effect: Colors in darker environments are perceived as less saturated than those in brighter environments (Hunt, 1950).

In Experiment 2, we examined the statistical differences between morning and evening depictions based on both human perception and metadata. We found that there are subtle differences between morning and evening depictions in terms of luminance but more significant differences in terms of chromaticity. These differences were particularly pronounced in perception-classified paintings, as opposed to the metadata-classified paintings. People tended to associate paintings depicting morning with a CCT similar to the average daylight of D55, while they perceived paintings depicting evening as having a CCT that ranges from warm to cool white. In addition, the airlight color of morning paintings was also bluer than that of evening paintings. One contributing factor to this differentiation might be the presence of depicted haze, which might serve as a visual cue in distinguishing between morning and evening scenes. These regularities used by observers to distinguish between morning and evening reflect recorded measurements of natural illumination. As the sun rises and solar elevation increases, the temperature transitions from low to high and the frequency of dense water vapor, haze, and fog tends to be higher in the morning than in the evening (Deutsch, 1991; Minnis et al., 1997; Pechony et al., 2007; Rickel & Genin, 2005). The presence of visible mist or haze in a scene can cause a higher level of diffuseness and lower color differences within the light field, resulting in an overall more even distribution of white in the image. The atmospheric filtering is also far from neutral (Lee & Hernández-Andrés, 2005; Yu, Wijntjes, Eisemann, & Pont, 2023), causing a blue-shifted airlight color in the morning relative to the evening. On the other hand, at sunset, the temperature decreases as the sun's elevation decreases, resulting in a higher proportion of water molecules in the lower atmosphere compared to the morning. These water molecules are larger than air molecules and scatter the long-wavelength component of sunlight, along with blue scattered light from the upper levels of the atmosphere, leading to large spatial and angular color variations in the sunset sky ranging from orangey-red to deep blue (Panorgias et al., 2012).

**Figure A4.**
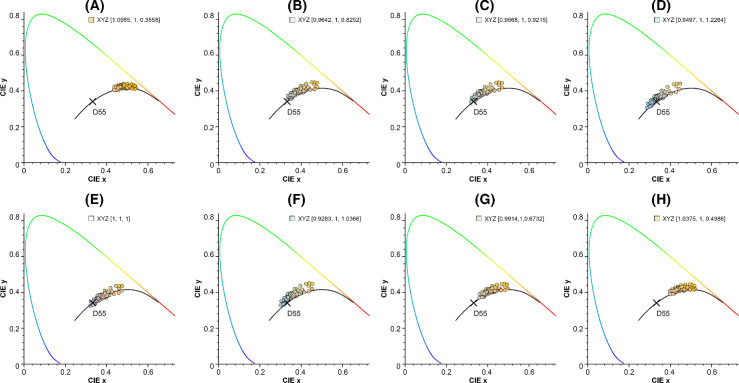
Results of Experiment 1. Mean image chromaticities in the CIE xy plane for paintings with a range of different white points, one disk per image. The disk colors represent the chromaticity of each image. The black line indicates the daylight locus and the location of D55 is marked. (**A**) Illuminant A. (**B**) D50. (**C**) D55. (**D**) D75. (**E**) Illuminant E. (**F**) Illuminant F1. (**G**) Illuminant F2. (**H**) Illuminant F3. The XYZ color coordinates of each white point are indicated in the chromaticity diagram.

It is worth noting that the reliability of metadata is an important consideration in this study. Certain forms of metadata may be based on the interpretation and knowledge of curators and might not necessarily reflect the original intentions of the painter. This is especially relevant for paintings created before the 19th century, when metadata were not yet being systematically recorded. However, most of the paintings used in this study were from the 19th century (Figure A4), and the primary metadata we used here were painting titles, which were generally chosen by the artists themselves.

**Figure A5.**
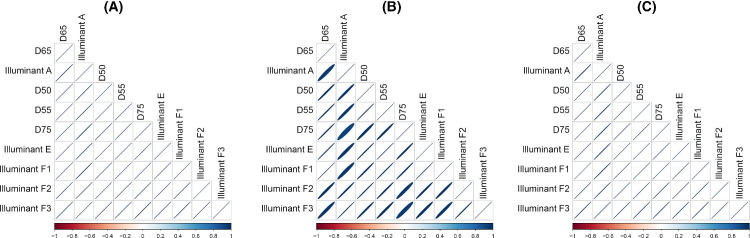
Results of Experiment 1. Correlation matrices for mean image luminance and chromaticity values in terms of CIE-Y and CIELAB a* and b* for nine different white points. Ellipses varying in thickness and color represent the correlation strength, with thinner ellipses indicating stronger correlations and fatter ellipses indicating weaker ones. The elongation of the ellipses shows the correlation direction, with red indicating positive, blue indicating negative, and white indicating no correlation. Matrix cells only include significant correlation coefficients at *p* < 0.05. (**A**) Luminance. (**B**) a* value. (**C**) b* value.

The chromaticity of a painting, calculated from the conversion of sRGB to XYZ values, may reflect the range of estimated illumination chromaticities that observers see in the painting under the assumption of a “gray world” (Buchsbaum, 1980). It is worth noting that the spread of chromaticities was calculated from the RGB values of the image, which may not produce the same chromaticity on every monitor due to variations in monitor specifications. To account for this, we can calculate the spread of chromaticities for a range of different white points, or assumed chromaticities for RGB values of [1, 1, 1]. While the transformation of the spread to different regions of the chromaticity diagram may vary, the relationship between chromaticity and time-of-day perception remains similar (see Figure A5).

In addition to the confounding factors of the actual colors of the painting and the colors displayed on participants’ screens, there may be a discrepancy between the artist's intended colors and the current colors due to color degradation. One influencing factor is the yellowing of varnish, which can affect the overall color palette of a painting and potentially influence the perceived time of day. Varnish serves as a protective layer on oil paintings, shielding them from environmental factors like dust, UV light, and moisture. While it is essential for preserving artwork and frequently employed in art restoration, the yellowing of varnish might alter perceptions of the time of day depicted, with yellow-tinted paintings possibly being seen as morning scenes. However, our experiments did not substantiate this hypothesis. Experiment 1 revealed a negative correlation between average time-of-day scores and the year of creation, with a significance level of *p* = 0.0051 (*r* = −0.27), indicating a weak correlation. However, a positive correlation would be expected if yellowing were occurring and influencing time-of-day judgments. In Experiment 2, the correlation was not statistically significant (*r* = −0.16, *p* = 0.1284). Additionally, the canvas ground used in the 19th century was lighter compared to those employed in earlier periods. This led to the hypothesis that darker paintings from earlier periods might be more frequently perceived as evening scenes. However, the correlations between average scores and the year of creation were either not statistically significant or very weak in both experiments.

In addition to creating a sense of space, our study shows that light and color in paintings are also associated with a temporal dimension: time of day in paintings. Specifically, we found that luminance and chromaticity are the most effective predictors of perceived time of day in paintings. Our findings show that image statistics might partly explain time-of-day perception in paintings.

## Conclusions

In this study, we analyzed the image statistics of paintings in order to understand how people perceive the time of day depicted in these works of art. We used dimension-reduction techniques to reduce the number of image statistics and then used these statistics to predict the perceived time of day in the paintings.

In conclusion, our study showed that the image statistics of paintings varied systematically depending on the time of day depicted, reflecting the characteristics of terrestrial illumination. Two predictors—luminance-related and chromaticity-related components—were the most effective at predicting the perceived time of day in the paintings. This suggests that people are able to perceive the difference between different time-of-day depictions in paintings and use cues related to luminance and chromaticity to discern the time of day depicted. Our results also indicate that while subtle and insignificant differences exist between morning and evening depictions in terms of luminance, statistically significant differences are evident in chromaticity. These average chromaticity differences appeared more pronounced in people's perceptions of the two times of day in paintings, rather than in metadata classification. We found that chromaticity may be an influential factor in people's perceptions of morning and evening and that observers can use both luminance and chromaticity to differentiate between the two times of day. These findings provide insight into color statistics of paintings that contribute to their perceived time of day and may be useful for artists and researchers studying the representation of the time of day in art.

## Appendix Appendix

### A1. Image color conversion and statistical estimation

The color of each pixel in a given image at position (1 ≤ *i* ≤ *W*, 1 ≤ *j* ≤ *H*) is described as a triplet of color coordinates in the sRGB color space as [R(i, j), G(i, j), B(i, j)], where *W* is the width and *H* is the height of the image in pixel number.

To ensure robust statistical estimates for the natural images, we excluded pixel values below the 5th percentile and above the 95th percentiles of the histograms, mitigating the influence of outlier pixel values that could represent random noise or artifacts within the image data.

Color conversions were as follows:

1. sRGB → XYZ and Yxy

The sRGB component values, R(*i*, *j*), G(*i*, *j*), and B(*i*, *j*), range from 0 to 1. The linear values, *C_linear_*(*i*, *j*), are calculated as follows:

$$\begin{array}{ccc} & & C_{linear}\left(i,j\right)=\\ & & \left\{\begin{array}{c} \frac{C_{sRGB}\left(i,j\right)}{12.92}\;,\;C_{sRGB}\left(i,j\right)\leq 0.04045\\ {\left(\frac{C_{sRGB}\left(i,j\right)\;+\;0.055}{1.055}\right)}^{2.4}\;,\;C_{sRGB}\left(i,j\right)>0.04045 \end{array}\right. \end{array}$$

where *C*(*i*, *j*) refers to R(*i*, *j*), G(*i*, *j*), or B(*i*, *j*).

Assuming standard chromaticity values for the R, G, and B components and a D65 white point, the conversion matrix from linear RGB to CIE 1931 tristimulus values is

$$\begin{array}{c} \;\left[\begin{array}{c} X\left(i,j\right)\\ Y\left(i,j\right)\\ Z\left(i,j\right) \end{array}\right]=\left[\begin{array}{ccc} 0.4124 & 0.3576 & 0.1805\\ 0.2126 & 0.7152 & 0.0722\\ 0.0193 & 0.1192 & 0.9503 \end{array}\right]\left[\begin{array}{c} R_{linear}\left(i,j\right)\\ G_{linear}\left(i,j\right)\\ B_{linear}\left(i,j\right) \end{array}\right] \end{array}$$

The CIE chromaticity coordinates (x, y) are derived from the tristimulus values (X, Y, Z) as follows:

$$\begin{array}{c} x\left(i,j\right)=\frac{X\left(i,j\right)}{X\left(i,j\right)+Y\left(i,j\right)+Z\left(i,j\right)} \end{array}$$

$$\begin{array}{c} y\left(i,j\right)=\frac{Y\left(i,j\right)}{X\left(i,j\right)+Y\left(i,j\right)+Z\left(i,j\right)} \end{array}$$

2. RGB → LMS / rod / melanopic receptoral activations

The spectrum of a given pixel is estimated as

$$\begin{array}{ccc} & & \lambda_{RGB}\left(i,j\right)\\ & & \;=\lambda_{R}\times R\left(i,j\right)+\lambda_{G}\times G\left(i,j\right)+\lambda_{B}\times B\left(i,j\right), \end{array}$$

where λ_*R*_ is the precomputed spectrum of the sRGB R primary (Mallett & Yuksel, 2019) and *R*(*i*, *j*) the linear R pixel value (and respectively for G and B).

The spectral sensitivity *S_k_*(*λ*) of the *k*th receptor type is specified by the Stockman and Sharpe (2000) cone fundamentals for L, M, and S; the melanopsin curve specified by Lucas et al. (2014) for melanopic irradiance; and the Crawford (1949) method for scotopic irradiance. The receptor-specific irradiance (*e_k_*) of a pixel can be calculated as follows:

$$\begin{array}{c} e_{k}\left(i,j\right)=\int \lambda_{RGB}\left(i,j\right)S_{k}\left(\lambda \right)d\lambda . \end{array}$$

3. XYZ → CIELAB

L^*^, a^*^, b^*^ quantities defined by the equations

$$\begin{array}{c} L^{*}\left(i,j\right)=116f\left(\frac{Y\left(i,j\right)}{Y_{n}}\right)-16 \end{array}$$

$$\begin{array}{c} a^{*}\left(i,j\right)=500\left[f\left(\frac{X\left(i,j\right)}{X_{n}}\right)-f\left(\frac{Y\left(i,j\right)}{Y_{n}}\right)\right] \end{array}$$

$$\begin{array}{c} b^{*}\left(i,j\right)=200\left[f\left(\frac{Y\left(i,j\right)}{Y_{n}}\right)-f\left(\frac{Z\left(i,j\right)}{Z_{n}}\right)\right] \end{array}$$

where

$$\begin{array}{ccc} & & f\left(X\left(i,j\right)/X_{n}\right)={\left(X\left(i,j\right)/X_{n}\right)}^{1/3}\\ & & \mathrm{if}\;X\left(i,j\right)/X_{n}<{\left(24/116\right)}^{3}\\ & & f\left(X\left(i,j\right)/X_{n}\right)=\left(\frac{841}{108}\right)\left(X\left(i,j\right)/X_{n}\right)+16/116\\ & & \mathrm{if}\;X\left(i,j\right)/X_{n}\geq {\left(24/116\right)}^{3} \end{array}$$

and

$$\begin{array}{ccc} & & f\left(Y\left(i,j\right)/Y_{n}\right)={\left(Y\left(i,j\right)/Y_{n}\right)}^{1/3}\\ & & \mathrm{if}\;Y/Y_{n}<{\left(24/116\right)}^{3} \end{array}$$

and

$$\begin{array}{ccc} & & f\left(Z\left(i,j\right)/Z_{n}\right)={\left(Z\left(i,j\right)/Z_{n}\right)}^{1/3}\\ & & \mathrm{if}\;Z/Z_{n}<{\left(24/116\right)}^{3} \end{array}$$

*X_n_*, *Y_n_*, *Z_n_* describe a specified white achromatic reference illuminant.

CIELAB lightness: L* as defined above
CIELAB chroma: *C** = arctan(*b**/*a**)
CIELAB hue: *h* = arctan(*b**/*a**)

Saturation is the degree to which a color is pure and is defined as the ratio of chroma to luminance.

$$\begin{array}{c} S\left(i,j\right)=\frac{C^{*}\left(i,j\right)}{L^{*}\left(i,j\right)} \end{array}$$

4. CCT (*T*):

The CCT of a given pixel *T*(*i*, *j*) is derived from its chromaticity in the (*u*, *v*) plane, in turn derived from (*x*, *y*) chromaticity in the usual way (Carter et al., 2018). We used a combination of triangular and parabolic calculations to estimate CCT from (*u*, *v*), reducing the error to 1 K (Ohno, 2014). We employed the triangular solution for |*D_uv_*| < 0.002 and the parabolic solution for other regions. By employing this method, we accurately estimated the CCT of each pixel, another measure of pixel chromaticity.

To offer a more uniform perceptual representation, we calculated the inverse CCT as 10^6^/CCT in reciprocal mega-Kelvin (MK^−1^). We denoted the inverse CCT as *T*′(*i*, *j*), where

$$\begin{array}{c} T^{'}\left(i,j\right)=\frac{10^{6}}{T\left(i,j\right)} \end{array}$$

The luminance-weighted CCT, denoted as *T_Y_*(*i*, *j*), is calculated as the product of the luminance *Y*(*i*, *j*) and CCT *T*(*i*, *j*):

The luminance-weighted CCT is given by the following:

$$\begin{array}{c} T_{Y}\left(i,j\right)=Y\left(i,j\right)\times \;T\left(i,j\right) \end{array}$$

5. The following formulae are used to calculate several statistical measures of an image, including the mean, minimum, maximum, variance, standard deviation, skewness, difference, and RMS contrast, based on the individual pixel data points represented by *r*(*i*, *j*):

Mean (µ):

$$\begin{array}{c} \mu \;=\frac{1}{WH}\sum_{i=1}^{W}\sum_{j=1}^{H}r\left(i,j\right) \end{array}$$

Minimum (min):

$$\begin{array}{c} r_{min}=\min_{\left(1\leq i\leq W;1\leq j\leq H\right)}r\left(i,j\right) \end{array}$$

Maximum (max):

$$\begin{array}{c} r_{max}=\max_{\left(0\leq i\leq W;0\leq j\leq H\right)}r\left(i,j\right) \end{array}$$

Variance (var):

$$\begin{array}{c} var\;=\;\frac{1}{WH}\sum_{i=1}^{W}\sum_{j=1}^{H}{\left[r\left(i,j\right)\;-\;\mu \right]}^{2} \end{array}$$

Standard deviation (σ):

$$\begin{array}{c} \sigma \;=\;\sqrt{var} \end{array}$$

Skewness (g):

$$\begin{array}{c} g=\frac{1}{WH}\frac{1}{{\left[\sqrt{var}\right]}^{3}}\sum_{i=1}^{W}\sum_{j=1}^{H}{\left[r\left(i,j\right)-\mu \right]}^{3} \end{array}$$

Difference (Δr):

$$\begin{array}{c} \Delta r=r_{max}-r_{min} \end{array}$$

RMS contrast (C):

$$\begin{array}{c} C=\sqrt{\frac{1}{WH}\sum_{i=1}^{W}\sum_{j=1}^{H}{\left[r\left(i,j\right)-\mu \right]}^{2}} \end{array}$$

6. The Euclidean image distance and correlation coefficient between two color channels of an image can be described using the following formulae, where *u* and *v* represent arbitrary color channels:

The Euclidean image distance:

$$\begin{array}{c} D_{uv}=\frac{1}{WH}\sqrt{\sum_{i=1}^{W}\sum_{j=1}^{H}{\left[u\left(i,j\right)-v\left(i,j\right)\right]}^{2}} \end{array}$$

This formula calculates the root mean square of the differences between corresponding pixel values in the two color channels over the entire image. It can be used to quantify the dissimilarity between the two color channels.

The correlation coefficient:

$$\begin{array}{c} R_{uv}=\frac{\sum_{i=1}^{W}\sum_{j=1}^{H}\left[u\left(i,j\right)-\bar{u}\right]\left[v\left(i,j\right)-\bar{v}\right]}{\sqrt{\sum_{i=1}^{W}\sum_{j=1}^{H}{\left[u\left(i,j\right)-\bar{S}\right]}^{2}{\left[v\left(i,j\right)-\bar{v}\right]}^{2}}} \end{array}$$

This formula calculates the correlation between the corresponding pixel values in the two channels over the entire image. It can be used to quantify the similarity between the two color channels. This measure can be useful in a variety of image-processing applications, such as color-based segmentation and image retrieval.

7. Color difference (ΔE_00_)

The color-difference formula (CIEDE2000) is based on the LAB color space.

$$\begin{array}{c} \Delta L{}^{'}=L_{max}^{*}-L_{min}^{*} \end{array}$$

$$\begin{array}{c} \bar{L}=\frac{L_{max}^{*}+L_{min}^{*}}{2} \end{array}$$

$$\begin{array}{c} \bar{C}=\frac{C_{max}^{*}+C_{min}^{*}}{2} \end{array}$$

$$\begin{array}{c} a_{max}^{'}=a_{max}^{*}+\frac{a_{max}^{*}}{2}\left(1-\sqrt{\frac{{\bar{C}}^{7}}{{\bar{C}}^{7}+25^{7}}}\right) \end{array}$$

$$\begin{array}{c} a_{min}^{'}=a_{min}^{*}+\frac{a_{min}^{*}}{2}\left(1-\sqrt{\frac{{\bar{C}}^{7}}{{\bar{C}}^{7}+25^{7}}}\right) \end{array}$$

$$\begin{array}{ccc} & & {\bar{C}}^{'}=\frac{C_{max}^{'}+C_{min}^{'}}{2}\;\text{and}\;\Delta C^{'}=C_{min}^{'}-C_{max}^{'}\\ & & \;\text{where}\;\\ & & C_{max}^{'}=\sqrt{a{{}_{max}^{'}}^{2}+b{{}_{max}^{*}}^{2}}{\;C}_{min}^{'}=\sqrt{a{{}_{min}^{'}}^{2}+b{{}_{min}^{*}}^{2}} \end{array}$$

$$\begin{array}{ccc} & & h{{}^{'}}_{max}=atan2\left({b^{*}}_{max},\;a{}_{max}^{'}\right)\;\;360^{\circ },\;\\ & & h{}_{min}^{'}=atan2\left({b^{*}}_{min},\;a{}_{min}^{'}\right)\;\;360^{\circ } \end{array}$$

$$\begin{array}{ccc} & & \Delta h^{'}=\\ & & \left\{\begin{array}{c} h_{min}^{'}-h_{max}^{'}\\ h_{min}^{'}-h_{max}^{'}+360^{\circ }\\ h_{min}^{'}-h_{max}^{'}-360^{\circ } \end{array}\right.\;\begin{array}{c} \left|h_{max}^{'}-h_{min}^{'}\right|\leq 180^{\circ }\\ \left|h_{max}^{'}-h_{min}^{'}\right|>180^{\circ },\;h_{min}^{'}\leq h_{max}^{'}\;\\ \left|h_{max}^{'}-h_{min}^{'}\right|>180^{\circ },\;h_{min}^{'}>h_{max}^{'}\; \end{array} \end{array}$$

$$\begin{array}{c} \Delta H^{'}=2\sqrt{C_{max}^{'}C_{min}^{'}}\sin\left(\Delta h^{'}/2\right) \end{array}$$

$$\begin{array}{ccc} & & {\bar{H}}^{'}=\\ & & \left\{\begin{array}{c} \left(h_{min}^{'}+h_{max}^{'}\right)/2\\ (h_{min}^{'}+h_{max}^{'}+360^{\circ })/2\\ \left(h_{min}^{'}+h_{max}^{'}-360^{\circ }\right)/2 \end{array}\right.\;\begin{array}{c} \left|h_{max}^{'}-h_{min}^{'}\right|\leq 180^{\circ }\\ \left|h_{max}^{'}-h_{min}^{'}\right|>180^{\circ },\;h_{min}^{'}+h_{max}^{'}<360^{\circ }\;\\ \left|h_{max}^{'}-h_{min}^{'}\right|>180^{\circ },\;h_{min}^{'}+h_{max}^{'}\geq 360^{\circ }\; \end{array} \end{array}$$

$$\begin{array}{ccc} T & = & 1-0.17\cos\left({\bar{H}}^{'}-30^{\circ }\right)+0.24\cos\left(2{\bar{H}}^{'}\right)\\ & & +\;0.32\cos\left(3{\bar{H}}^{'}+6^{\circ }\right)-0.20\cos\left(4{\bar{H}}^{'}+63^{\circ }\right) \end{array}$$

$$\begin{array}{ccc} & & S_{L}=1+\frac{0.015{\left(\bar{L}-50\right)}^{2}}{\sqrt{20+{\left(\bar{L}-50\right)}^{2}}}\;\\ & & S_{C}=1+0.045{\bar{C}}^{'}\;S_{H}=1+0.015{\bar{C}}^{'}T \end{array}$$

$$\begin{array}{ccc} R_{T} & = & \\ & & \;-\;2\;\sqrt{\frac{{\bar{C}}^{{'}^{7}}}{{\bar{C}}^{{'}^{7}}+25^{7}}\sin\;\left[\;60^{\circ }\cdot \exp\;\left(\;-{\left[\frac{{\bar{H}}^{'}-275^{\circ }}{25^{\circ }}\right]}^{2}\right)\;\right]} \end{array}$$

$$\begin{array}{c} K_{L}=K_{C}=K_{H}=1 \end{array}$$

$$\begin{array}{ccc} & & \;\Delta E_{00}\left(L_{max}^{*},a_{max}^{*},b_{max}^{*};L_{min}^{*},a_{min}^{*},b_{min}^{*}\right)\\ & & \;=\sqrt{{\left(\frac{\Delta L{}^{'}}{K_{L}S_{L}}\right)}^{2}+{\left(\frac{\Delta C{}^{'}}{K_{C}S_{C}}\right)}^{2}+{\left(\frac{\Delta H{}^{'}}{K_{H}S_{H}}\right)}^{2}+R_{T}\left(\frac{\Delta C{}^{'}}{K_{C}S_{C}}\right)\left(\frac{\Delta H{}^{'}}{K_{H}S_{H}}\right)} \end{array}$$

The color difference between the mean of 5% brightest pixels and the mean of 5% darkest pixel is

$$\begin{array}{c} \Delta E_{00}=\Delta E_{00}\left(L_{max}^{*},a_{max}^{*},b_{max}^{*};L_{min}^{*},a_{min}^{*},b_{min}^{*}\right) \end{array}$$

8. Mean image colorfulness (C)

*rg* is the difference between the R channel and the G channel. *yb* represents half of the sum of the R and G channels minus the B channel.

$$\begin{array}{ccc} rg & \;= & R-G\\ yb & \;= & \frac{1}{2}\left(R+G\right)-B \end{array}$$

Next, the standard deviation (*σ_rgyb_*) and mean (*µ_rgyb_*) are computed before calculating the final colorfulness metric, C.

$$\begin{array}{c} \mu_{rg}=\frac{1}{WH}\sum_{i=1}^{W}\sum_{j=1}^{H}rg\left(i,j\right) \end{array}$$

$$\begin{array}{c} \mu_{yb}=\frac{1}{WH}\sum_{i=1}^{W}\sum_{j=1}^{H}yb\left(i,j\right) \end{array}$$

$$\begin{array}{c} \sigma_{rg}=\sqrt{\frac{1}{WH}\sum_{i=1}^{W}\sum_{j=1}^{H}{\left[rg\left(i,j\right)-\bar{rg}\right]}^{2}} \end{array}$$

$$\begin{array}{c} \sigma_{yb}=\sqrt{\frac{1}{WH}\sum_{i=1}^{W}\sum_{j=1}^{H}{\left[yb\left(i,j\right)-\bar{yb}\right]}^{2}} \end{array}$$

$$\begin{array}{c} \sigma_{rgyb}=\sqrt{{\sigma_{rg}}^{2}+{\sigma_{yb}}^{2}} \end{array}$$

$$\begin{array}{c} \mu_{rgyb}=\sqrt{{\mu_{rg}}^{2}+{\mu_{yb}}^{2}} \end{array}$$

The mean image colorfulness is given by the following:

$$\begin{array}{c} C=\sigma_{rgyb}+0.3*\mu_{rgyb} \end{array}$$

9. Luma

The luma *Y*′(*i*, *j*) of a pixel in an image is calculated as a linear combination of its RGB primary values and is commonly used in color video encoding in addition to luminance due to its colorimetric properties. The formula for *Y*′(*i*, *j*) is as follows:

$$\begin{array}{ccc} Y{}^{'}\left(i,j\right) & \;= & 0.2126R\left(i,j\right)+0.7152G\left(i,j\right)\\ & & +\;0.0722B\left(i,j\right) \end{array}$$

10. Airlight estimation

A practical way to estimate airlight color comes from dehazing literature, as airlight estimation is a prerequisite for dehazing. In a commentary on the dehazing study by He et al. (2009), Odisio and Alessandrini (2014) outline the following way of airlight estimation:

(1) First apply a filter to each RGB channel that replaces each pixel value with the local minimum value, defined by some kernel width *r*. This step is used for local smoothing.
(2) For each pixel, take the minimum value of three channels, resulting in a dark channel image.
(3) Select the top 0.3% of the brightest pixels of the dark channel.
(4) Cluster these pixels and select the cluster with the highest luminance.
(5) Take the mean RGB color, and this is the airlight.

### A2. Correlation matrices of human rating and image statistics

In Figure A1 and Figure A2, we illustrate the correlations between human ratings and selected image statistics for Experiments 1 and 2, resulting from independent regressions of mean rating scores on each image statistics measure. The red time-of-day score corresponds to the mean score, which is the sum of all scores divided by the number of scores, representing an overall measure of the perceived time of day for each painting. The morningness score, noonness score, eveningness score, and nightness score represent the proportional score per category, indicating the proportion of scores falling into each category relative to the total count of scores.

### A3. Principal component analysis: factor loadings and explained variance for Experiment 1

Table A1 displays the factor loadings for the first five principal components of paintings in Experiment 1. The table shows the loading values for each image statistic, with red indicating negative loading and green indicating positive loading.

**Table A1.** Results of Experiment 1. *Notes*: Factor loadings for the first five principal components. Extraction method: principal component analysis. ^a^Five components extracted.

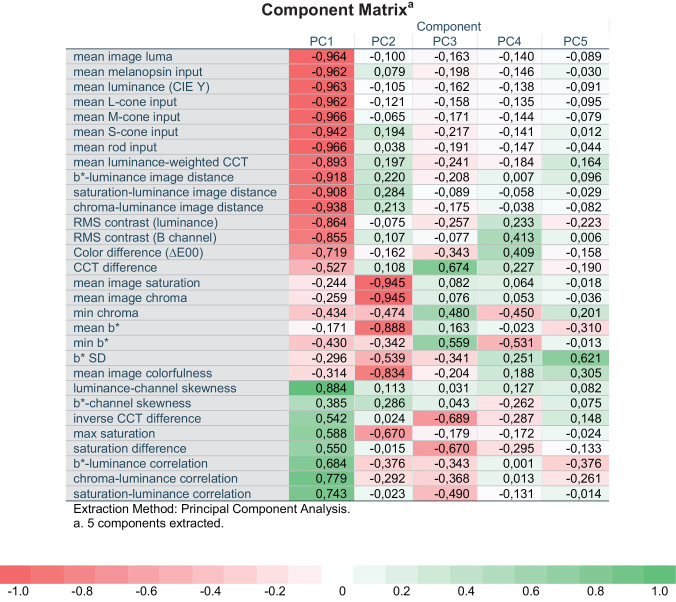

Table A2 elaborates on the results from Experiment 1, providing a detailed breakdown of the total variance explained by each of the principal components. The components are listed in descending order of the variance they account for. For each principal component, the table presents the initial eigenvalues, the percentage of total variance explained, and the cumulative percentage of variance explained up to that component. Notably, the first few components explain a substantial proportion of the total variance, indicating their significant contribution in capturing the patterns in the image data.

**Table A2.** Results of Experiment 1, showing the total variance explained by each principal component in the analysis. *Notes*: The components are ordered by the amount of variance they explain, from highest to lowest. Extraction method: principal component analysis.

Total variance explained						
	Initial eigenvalues			Extraction sums of squared loadings		
Component	Total	% of variance	Cumulative %	Total	% of variance	Cumulative %
1	16.292	54.307	54.307	16.292	54.307	54.307
2	5.027	16.758	71.065	5.027	16.758	71.065
3	3.205	10.682	81.747	3.205	10.682	81.747
4	1.514	5.046	86.793	1.514	5.046	86.793
5	1.069	3.563	90.355	1.069	3.563	90.355
6	0.894	2.979	93.334			
7	0.448	1.492	94.826			
8	0.347	1.156	95.982			
9	0.280	0.934	96.916			
10	0.181	0.602	97.518			
11	0.170	0.566	98.084			
12	0.132	0.439	98.523			
13	0.104	0.347	98.870			
14	0.078	0.262	99.132			
15	0.074	0.248	99.380			
16	0.049	0.164	99.544			
17	0.045	0.149	99.693			
18	0.035	0.117	99.810			
19	0.022	0.073	99.883			
20	0.016	0.054	99.937			
21	0.012	0.039	99.976			
22	0.004	0.014	99.990			
23	0.002	0.008	99.998			
24	0.000	0.001	99.999			
25	0.000	0.001	100.000			
26	8.233E-5	0.000	100.000			
27	9.315E-16	3.105E-15	100.000			
28	2.195E-16	7.316E-16	100.000			
29	1.842E-18	6.141E-18	100.000			
30	–1.278E-15	–4.258E-15	100.000			

### A4. Distribution of years of creation for paintings in Experiments 1 and 2

Figure A3 shows the distribution of years of creation for the paintings used in Experiments 1 and 2. The histogram demonstrates that most of the paintings were created around 1900, with relatively fewer paintings from earlier or later periods. This information is helpful in understanding the potential reliability of metadata.

### A5. Mean chromaticities of paintings for varying screen white points

To account for the potential impact of different white points on the calculation of chromaticities, we calculated the spread of mean image chromaticities for Experiment 1 for a range of different white points, or assumed chromaticities for RGB values of [1, 1, 1]. This allowed us to consider the effect of chromatic adaptation or different monitor white points on the calculation of chromaticities in our study. In Figure A4, we plot the mean image chromaticities in the CIE xy plane for a range of different white points, with one disk representing one image.

To further investigate the relationship between luminance and chromaticity across different white points, we plotted the correlation matrices of mean image luminance, CIELAB a*, and CIELAB b* values for nine different white points in Figure A5. We found that both luminance and chromaticity are significantly and strongly correlated across different white points, while the correlation of CIELAB a* is relatively lower. This is as expected, given that the mean image chromaticity follows a distinct pattern along the daylight locus with only minor green shift.
